# Impact of simplified HCV diagnostic strategies on the HCV epidemic among men who have sex with men in the era of HIV oral pre‐exposure prophylaxis in Taiwan: a modelling study

**DOI:** 10.1002/jia2.26251

**Published:** 2024-05-02

**Authors:** Huei‐Jiuan Wu, Sophy TingFang Shih, Tanya L. Applegate, Jisoo A. Kwon, Evan B. Cunningham, Jason Grebely, Richard T. Gray

**Affiliations:** ^1^ The Kirby Institute UNSW Sydney New South Wales Australia; ^2^ Department of Public Health College of Medicine National Cheng Kung University Tainan Taiwan

**Keywords:** point‐of‐care testing, hepatitis C, dried blood spot testing, point‐of‐care RNA testing, clinic‐based reflex RNA testing, HIV pre‐exposure prophylaxis

## Abstract

**Introduction:**

Simplified hepatitis C virus (HCV) diagnostic strategies have the potential to improve HCV diagnoses and treatment. We aimed to investigate the impact of simplified HCV diagnostic strategies on HCV incidence and its effect on HCV diagnosis and treatment among men who have sex with men (MSM) regardless of HIV status and use of HIV pre‐exposure prophylaxis (PrEP) in Taiwan.

**Methods:**

A compartmental deterministic model was developed to describe the natural history of HCV disease progression, the HCV care cascade and the HIV status and PrEP using among MSM. The model was calibrated to available data for HCV and HIV epidemiology and population demographics in Taiwan. We simulated the epidemic from 2004 and projected the impact of simplified testing strategies on the HCV epidemic among MSM over 2022–2030.

**Results:**

Under the current testing approach in Taiwan, total HCV incidence would increase to 12.6 per 1000 person‐years among MSM by 2030. Single‐visit point‐of‐care RNA testing had the largest impact on reducing the number of new HCV infections over 2022–2030, with a 31.1% reduction (interquartile range: 24.9%−32.8%). By 2030, single‐visit point‐of‐care HCV testing improved HCV diagnosis to 90.9%, HCV treatment to 87.7% and HCV cure to 81.5% among MSM living with HCV. Compared to status quo, prioritized simplified HCV testing for PrEP users and MSM living with diagnosed HIV had considerable impact on the broader HCV epidemic among MSM. A sensitivity analysis suggests that reinfection risk would have a large impact on the effectiveness of each point‐of‐care testing scenario.

**Conclusions:**

Simplified HCV diagnostic strategies could control the ongoing HCV epidemic and improve HCV testing and treatment among Taiwanese MSM. Single‐visit point‐of‐care RNA testing would result in large reductions in HCV incidence and prevalence among MSM. Efficient risk‐reduction strategies will need to be implemented alongside point‐of‐care testing to achieve HCV elimination among MSM in Taiwan.

## 1 INTRODUCTION

Among men who have sex with men (MSM), hepatitis C virus (HCV) is transmitted via sexual contact and sharing of unsterile injecting equipment and disproportionately affects MSM living with HIV [1]. Recent data suggest that transmission of HCV spreads from MSM living with HIV to HIV‐negative MSM [2]. A higher HCV incidence has been observed in MSM receiving HIV pre‐exposure prophylaxis (PrEP) compared to HIV‐negative MSM not receiving PrEP [3]. It is suspected that increased uptake of PrEP for HIV prevention resulted in sexual network overlapping between HIV‐negative MSM and MSM living with HIV. Taiwan has one of the highest prevalence of HCV infection (3.5%) among overall MSM in the Western Pacific region, and HCV prevalence is substantially higher in MSM living with HIV (4.5%) than in HIV‐negative MSM (0.7%) [2]. Given a potentially expanding HCV epidemic from MSM living with HIV to HIV‐negative MSM, HCV prevention and treatment strategies targeted at MSM, regardless of their HIV status, are urgently needed to control the burden of HCV among MSM in Taiwan.

The high efficacy and safety of direct‐acting antiviral (DAA) treatments (efficacy above 90%) provide an opportunity to control the HCV epidemic [4, 5]. However, the complexity of the current diagnostic algorithm for HCV represents a barrier to diagnosis, treatment and care, with an estimated 63% of people living with HCV remaining undiagnosed globally [6]. In Taiwan, an estimated 25% of MSM living with HIV and HCV do not have access to HCV care and treatment despite unrestricted access to DAA treatments since 2019 [6]. The implication is that a higher percentage of HIV‐negative MSM living with HCV do not access HCV care due to less HIV engagement with healthcare than MSM living with HIV.

A simplified testing algorithm is desired to shorten the time from diagnosis to treatment initiation and facilitate the uptake of HCV DAAs [7]. Several testing strategies have demonstrated the potential to improve HCV diagnosis and treatment [7], including point‐of‐care testing, which allows testing to be conducted onsite with delivery of results in an hour, and could improve HCV diagnosis and treatment [8]. The use of point‐of‐care RNA testing has been shown to reduce the time from antibody testing to treatment initiation and increase treatment uptake compared to laboratory‐based standard‐of‐care HCV RNA testing [9]. Other simplified testing algorithms, such as clinic‐based reflex HCV RNA testing, which requires blood samples collected at a single visit, are used for both antibody and RNA testing if needed (reducing the need for several visits), and is recommended for key populations (particularly people who inject drugs and MSM) by the World Health Organization (WHO) [10]. Previous modelling has considered the impact of testing and treatment on the HCV epidemic among MSM living with HIV or MSM overall [11, 12, 13, 14, 15, 16, 17, 18]. However, previous research has not assessed and compared the impact of various simplified testing strategies on the HCV epidemic among MSM in the era of PrEP.

In this study, we use modelling to evaluate the impact of various simplified HCV testing strategies on HCV incidence, prevalence, and its effect on the HCV diagnosis, treatment and cure among MSM in Taiwan to determine which strategy would have the greatest impact on reducing HCV.

## 2 METHODS

**Figure 1:**
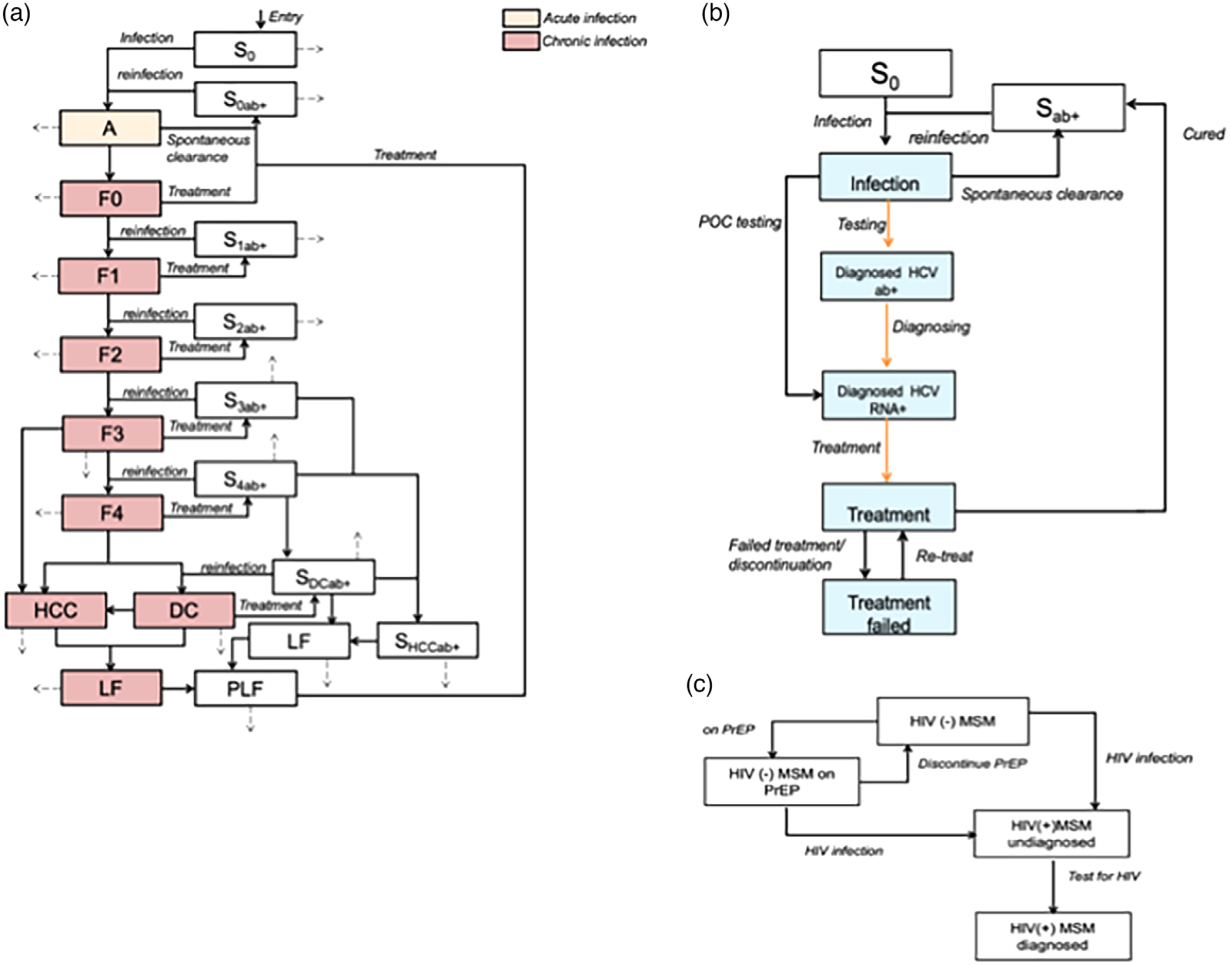
Model schematic. **(a)** HCV transmission and disease progression. Dashed arrows represent mortality. White blocks represent susceptible, including those spontaneously cleared and cured from HCV. **(b)** HCV care cascade and HCV testing pathways. Orange arrows indicate the movement influenced by the testing scenarios. **(c)** Population groups and transitions. A, acute HCV infection; DC, decompensated cirrhosis; F0−F4, chronic HCV infection classified by stage of liver fibrosis with METAVIR score; HCC, hepatocellular carcinoma; LF, liver failure and requires treatment; PLF, liver failure and received treatment; S, susceptible and never infected with HCV.

We developed a deterministic dynamic compartmental model to assess the HCV epidemic among MSM in Taiwan. The model included HCV infection, disease progression, cascades of care, transitions between MSM sub‐populations and mortality (background, HIV and HCV related) as shown in Figure 1. We calibrated the model to available population and HCV epidemiological data from Taiwan. We then simulated the epidemic from 2004 and projected the impact of simplified testing strategies on the HCV epidemic among MSM from 2022 to the end of 2030.

### 2.1 Model structure

We stratified the Taiwanese MSM population into four sub‐groups based on HIV infection, the status of PrEP use, and HIV treatment to reflect the differences in the HCV epidemic and the level of health services engagement, for example due to HIV treatment or PrEP use. MSM entered the modelled population susceptible to HIV and HCV infection as they become sexually active. This HCV transmission rate incorporated all the effects of the behaviours contributing to HCV acquisition and the level of contact between populations. Technical document for the model development is available on the GitHub repository associated with this project [19].

Once HCV infected, individuals entered undiagnosed acute HCV infection and either spontaneously cleared the infection and became susceptible again (but HCV antibody positive) or developed chronic HCV infection but at a different rate based on their HIV infection status to reflect the impact of HCV disease progression by HIV/HCV coinfection [20]. MSM with advanced liver disease (decompensated cirrhosis, hepatocellular carcinoma, liver failure and requires treatment, and liver failure and received treatment) had additional HCV‐related liver mortality, and HCV‐related liver mortality was reduced when they attained HCV cure but still elevated compared to the susceptible population [21, 22, 23].

In terms of parameters around the HCV cascade of care, the model incorporated the impact of DAA on treatment initiation and sustained virologic response (SVR) with increasing treatment initiation rate and SVR rate since 2017 to reflect the rollout of DAA treatment in Taiwan.

Treatment initiation probability for MSM living with diagnosed HIV was extracted from ATHENA cohort study, which is a national cohort study of people living with HIV in the Netherlands [24], due to the limited generalizability of Taiwan‐specific data from a single‐centre study [25]. Lower health service engagement for other sub‐populations was assumed, referencing the relative increase reported in the ATHENA study [24]. The detailed justification of parameters around the HCV cascade of care is provided in Table S1.

### 2.2 Model parameterization and calibration

Best estimates and uncertainty ranges for model parameters were informed by available literature with a preference of study setting in Taiwan and from Taiwan HCV policy guidelines. The uncertainty interval of parameters was obtained either from the literature or, if not available, set to an assumed plausible range. We used available demographic and epidemiological data to calibrate the model to reflect HCV epidemic among Taiwanese MSM over 2004–2020 and future trends up to 2030. Epidemiological data were obtained from the HIV database of Taiwan CDC, “Taiwan Hepatitis C Policy Guidelines 2018–2025” and published literature [26, 27, 28]. Further details of model description, parameters and calibration are provided in Section 1 of the Supplementary Material and Tables S1−S3.

### 2.3 Uncertainty analysis

Given the uncertainty in the data underlying the model's parameterization and calibration, we conducted a Monte Carlo uncertainty analysis with 1000 simulations to obtain a 2.5%−97.5% percentile range (95% percentile interval, 95% PI). We report results using the best estimates and 95% PI from the 1000 model simulations.

### 2.4 Simplified HCV diagnostic strategies

In Taiwan, current HCV diagnosis involves a two‐step laboratory test, with three clinical visits needed for treatment access. Since October 2021, all doctors can prescribe DAAs without a hepatologist referral. MSM living with HIV received integrated HCV testing at tertiary hospitals, combining liver clinics, infectious disease clinics and on‐site laboratories. As a result, the co‐locating laboratory and medical specialists have the flexibility to adopt a simplified HCV diagnostic algorithm and facilitate the uptake of HCV treatment. The simplified HCV testing strategies considered in our model included (1) point‐of‐care HCV antibody testing (people receiving testing in non‐clinical settings and two clinic visits required to initiate HCV treatment. To test the impact of expanding HCV screening into non‐clinical settings); (2) dried blood spot (DBS) testing (samples required to perform in a laboratory and two visits for HCV treatment initiation); (3) clinic‐based reflex RNA testing (sample collected at first visit for on‐site antibody testing and if returning a positive result would be immediately followed by a laboratory‐based HCV RNA testing. Two visits for HCV treatment initiation); and (4) point‐of‐care RNA testing (single visit for diagnosis and treatment initiation). Further details of the testing scenarios are provided in Section 2 of the Supplementary Material.

The effect of simplified HCV testing‐based interventions was captured in the model by changing the HCV antibody testing, HCV RNA testing and HCV treatment initiation rates. To estimate the effect of HCV testing in our scenarios, we extracted from a meta‐analysis the pooled odds ratios of testing interventions on the uptake of HCV antibody testing, HCV RNA testing and HCV treatment initiation compared with standard of care HCV testing [7]. We assumed the testing coverage within each population would saturate at 98% allowing the possibility of some loss to follow‐up and each simplified HCV testing strategy would replace the current HCV testing approach within a 2‐year period (2022−2024) to demonstrate the impact of a systematic scale‐up. The HCV testing and treatment parameters in each scenario are shown in Table 1. Parameter values, presented as annual probability, indicate higher uptake proportion accelerates the transition of individuals between stages.

**Table 1:** Parameterization of the simplified HCV testing strategies

Scenario	Sub‐populations	Anti‐HCV testing rate, %/year	HCV RNA testing rate, %/year	DAA initiation rate, %/year	Notes
Status quo	HIV negative not on PrEP	25.0% (18.8%−31.3%)	50.0% (37.5%−62.5%)	27.0% (20.2%−33.7%)	Reference scenario
	HIV negative on PrEP	25.0% (18.8%−31.3%)	50.0% (37.5%−62.5%)	27.0% (20.2%−33.7%)	
	HIV positive and undiagnosed	25.0% (18.8%−31.3%)	50.0% (37.5%−62.5%)	27.0% (20.2%−33.7%)	
	HIV diagnosed and on treatment	80.0% (60.0%−98.0%)	80.0% (60.0%−98.0%)	67.8% (50.9%−84.8%)	
Point‐of‐care antibody testing	HIV negative not on PrEP	74.8% (34.8%−98.0%)	50.0% (37.5%−62.5%)	27.0% (20.2%−33.7%)	1
	HIV negative on PrEP	74.8% (34.8%−98.0%)	50.0% (37.5%−62.5%)	27.0% (20.2%−33.7%)	
	HIV positive and undiagnosed	74.8% (34.8%−98.0%)	50.0% (37.5%−62.5%)	27.0% (20.2%−33.7%)	
	HIV diagnosed and on treatment	98.0% (98.0%−98.0%)	80.0% (60.0%−98.0%)	67.8% (50.9%−84.8%)	
Dried blood spot testing	HIV negative not on PrEP	52.3% (28.6%−98.0%)	89.0% (75.0%−98.0%)	27.0% (20.2%−33.7%)	2
	HIV negative on PrEP	52.3% (28.6%−98.0%)	89.0% (75.0%−98.0%)	27.0% (20.2%−33.7%)	
	HIV positive and undiagnosed	52.3% (28.6%−98.0%)	89.0% (75.0%−98.0%)	27.0% (20.2%−33.7%)	
	HIV diagnosed and on treatment	98.0% (98.0%−98.0%)	89.0% (75.0%−98.0%)	67.8% (50.9%−84.8%)	
Clinic‐based reflex RNA testing	HIV negative not on PrEP	75.3% (74.3%−98.0%)	89.0% (75.0%−98.0%)	27.0% (20.2%−33.7%)	2
	HIV negative on PrEP	75.3% (74.3%−98.0%)	89.0% (75.0%−98.0%)	27.0% (20.2%−33.7%)	
	HIV positive and undiagnosed	75.3% (74.3%−98.0%)	89.0% (75.0%−98.0%)	27.0% (20.2%−33.7%)	
	HIV diagnosed and on treatment	98.0% (98.0%−98.0%)	89.0% (75.0%−98.0%)	67.8% (50.9%−84.8%)	
Single‐visit point‐of‐care RNA testing	HIV negative not on PrEP	NA	36.8% (26.5%−56.0%)	36.2% (15.4%−67.4%)	3
	HIV negative on PrEP	NA	36.8% (26.5%−56.0%)	36.2% (15.4%−67.4%)	
	HIV positive and undiagnosed	NA	36.8% (26.5%−56.0%)	36.2% (15.4%−67.4%)	
	HIV diagnosed and on treatment	NA	98.0% (84.6%−98.0%)	90.9% (39.2%−98.0%)	

*Notes*: The HCV antibody testing rate is defined as the annual percentage of HCV‐infected people receiving antibody testing; the RNA testing rate is defined as the annual percentage of people who tested for HCV antibody testing receiving HCV RNA testing; and the DAA initiation rate is defined as the annual percentage of people who tested for HCV RNA testing initiating HCV DAA treatment. These annual percentages to receive HCV antibody testing, HCV RNA testing and initiate HCV DAA treatment are converted to a proportion in the model.

1. We assumed the same annual percentage of receiving HCV RNA testing as for the status‐quo scenario due to a lack of estimation for impact of POC antibody testing on HCV RNA testing.

2. We assumed the same annual percentage of HCV DAA treatment initiation as for the status‐quo scenario due to a lack of estimation for impact of POC antibody testing, DBS testing and POC reflex RNA testing on HCV DAA treatment initiation.

3. HCV antibody testing was not required in the POC RNA testing. We assumed POC annual percentage of receiving HCV RNA testing replaced current antibody testing and assumed the annual percentage of receiving RNA testing is the same as the annual percentage of receiving HCV antibody testing for the POC antibody testing strategy.

### 2.5 Sensitivity analysis

We conducted one‐way sensitivity analyses where we varied the following: (1) the scale‐up time frame of the HCV testing scenario (optimized scale‐up: 1 year vs. pessimistic scale‐up: 5 years); (2) the effect of simplified HCV testing strategy on improving HCV testing (optimized impact vs. pessimistic impact); and (3) HCV reinfection rate (1.5‐fold of primary infection rate vs. no HCV reinfection). We further explored simplified HCV testing strategies targeted at MSM on PrEP and MSM who are HIV diagnosed (and on treatment). (See Section 2 of the Supplementary Material.)

The base parameter values in the sensitivity analysis for each simplified HCV testing strategy were a 2‐year period (2022−2024) with the same HCV reinfection and primary infection rates. The base values of HCV antibody/RNA testing for each simplified HCV testing strategy and MSM subgroup are shown in Table 1.

### 2.6 Outcomes

We obtained estimates of HCV incidence, prevalence, number of people living with chronic HCV, number of HCV‐related deaths and the number of annual new HCV infections among MSM in Taiwan up to 2030 for each scenario. We defined HCV prevalence as a proportion of MSM living with HCV viraemia and HCV incidence as the number of annual new HCV infections. For each simplified HCV testing strategy, we calculated the relative change, compared with the reference scenario, in the cumulative number of new HCV infections between 2022 and 2030.

We also evaluated the impact of simplified HCV testing strategies on the proportion of people living with HCV diagnosed, treated and achieved SVR or were cured since the start of the DAA era in 2017. The proportion diagnosed each year was defined as the number of people who have been diagnosed and are still living since the end of 2016 at the end of the year divided by the number of people who have lived with chronic HCV since the end of 2016 (including those still living with HCV and those cured) at the end of the previous year [29]. The same calculation was applied to estimate the HCV treatment initiation and SVR coverage.

Model development, analyses and figure generation were conducted using R (version 4.2.1) [30, 31]. The code for the final model is publicly available under an open‐access licence [19]. No ethical approval or consent was required since this study used publicly available data only and data were accessed from January 2021 to December 2022.

## 3 RESULTS

Under current testing and treatment practice, we estimated the overall HCV prevalence increased from 1.9% (95% PI: 1.8%−1.9%) in 2004 to 4.5% (95% PI: 3.0%−5.6%) in 2022. A similar trend in HCV incidence was estimated with an increase from 1.4 per 1000 person‐years (95% PI: 1.1−1.8) in 2004 to 8.6 per 1000 person‐years (95% PI: 3.9−15.0) in 2022. The model projected an HCV prevalence of 5.5% (95% PI: 3.5%−6.5%) and incidence of 12.6 (95% PI: 5.3−20.6) per 1000 person‐years by 2030, respectively (Table 2). The model projected that 81.5% had been diagnosed, 71.3% of those had initiated treatment and 65.2% of those had been cured among people living with HCV (shown in Table 2 and Figure 3). In MSM subgroups, the HCV prevalence and incidence among MSM living with HIV regardless of the status of HIV diagnosis are higher than HIV‐negative MSM, which aligned with the HCV epidemic among MSM subgroups in Taiwan. The calibrated model is shown in Figures S2−S7.

**Table 2:** Summary table of results for simplified HCV testing strategies among MSM in Taiwan in 2030

POC testing scenario	Prevalence%	Incidence per 1000 PY	Number of people living with chronic HCV	Number of annual new infections	Number of annual HCV‐related deaths	Percentages of diagnosed, treatment initiated and cured
**Status quo**	5.5 (3.5−6.5)	12.6 (5.3−20.6)	10,249 (4463−12,303)	2561 (751−4342)	162 (100−294)	82%, 71%, 65%
**Base case**						
**Point‐of‐care antibody testing**	4.2 (2.3−7.9)	9.6 (3.6−24.4)	7761 (2744−13,836)	2083 (502−4822)	160 (98−288)	88%, 78%, 72%
**Dried blood spot testing**	3.8 (2.0−7.6)	8.7 (3.1−23.8)	6971 (2533−12,821)	1896 (438−4544)	157 (96−286)	90%, 81%, 74%
**Clinic‐based reflex RNA testing**	3.5 (1.9−7.2)	8.0 (3.0−22.7)	6404 (2397−12,182)	1740 (414−4465)	155 (95−284)	91%, 82%, 76%
**Point‐of‐care RNA testing**	2.5 (1.4−5.6)	5.9 (2.3−17.8)	4384 (1699−9436)	1284 (320−3082)	149 (91−275)	91%, 88%, 82%
**Pessimistic scale‐up (5 years)**						
**Point‐of‐care antibody testing**	4.5 (2.1−8.2)	10.1 (3.3−25.3)	8156 (3043−16,016)	2187 (463−4988)	161 (98−287)	88%, 77%, 71%
**Dried blood spot testing**	4.1 (2.1−7.8)	9.4 (3.3−24.6)	7508 (2672−13,616)	2036 (464−4841)	159 (97−287)	89%, 79%, 73%
**Clinic‐based reflex RNA testing**	3.8 (2.0−7.6)	8.7 (3.1−23.7)	7007 (2716−14,092)	1901 (447−4667)	158 (97−286)	90%, 81%, 74%
**Point‐of‐care RNA testing**	2.7 (1.5−3.7)	6.4 (2.4−12.7)	4763 (2814−13,273)	1391 (334−2480)	151 (92−274)	91%, 87%, 81%
**Optimized scale‐up (1 year)**						
**Point‐of‐care antibody testing**	4.1 (2.3−7.8)	9.4 (3.5−24.2)	7612 (2700−13,632)	2045 (495−4771)	159 (97−288)	88%, 79%, 72%
**Dried blood spot testing**	3.7 (1.9−7.4)	8.5 (3.0−23.2)	6789 (2478−12,476)	1848 (429−4450)	156 (96−284)	90%, 81%, 75%
**Clinic‐based reflex RNA testing**	3.4 (1.8−7.0)	7.7 (2.9−22.0)	6195 (2318−11,812)	1685 (403−4340)	154 (95−283)	91%, 83%, 76%
**Point‐of‐care RNA testing**	2.4 (1.3−5.5)	5.8 (2.2−17.6)	4267 (1654−9419)	1252 (312−3042)	148 (91−274)	91%, 88%, 82%
**Pessimistic impact on HCV diagnosis**						
**Point‐of‐care antibody testing**	5.0 (2.3−7.9)	11.2 (3.6−24.4)	9130 (2744−13,836)	2442 (502−4822)	164 (98−288)	85%, 74%, 68%
**Dried blood spot testing**	4.4 (2.0−7.6)	9.9 (3.1−23.8)	7954 (2533−12,821)	2160 (438−4544)	160 (96−286)	87%, 78%, 71%
**Clinic‐based reflex RNA testing**	4.4 (1.9−7.2)	9.9 (3.0−22.7)	7949 (2397−12,182)	2159 (414−4465)	160 (95−284)	87%, 78%, 71%
**Point‐of‐care RNA testing**	2.7 (1.4−5.6)	6.4 (2.3−17.8)	4744 (1699−9436)	1390 (320−3082)	150 (91−275)	90%, 87%, 80%
**Optimistic impact on HCV diagnosis**						
**Point‐of‐care antibody testing**	4.2 (2.3−7.9)	9.5 (3.6−24.4)	7668 (2744−13,836)	2059 (502−4822)	159 (98−288)	88%, 79%, 72%
**Dried blood spot testing**	3.4 (2.0−7.6)	7.9 (3.1−23.8)	6322 (2533−12,821)	1717 (438−4544)	155 (96−286)	91%, 82%, 76%
**Clinic‐based reflex RNA testing**	3.4 (1.9−7.2)	7.9 (3.0−22.7)	6318 (2397−12,182)	1716 (414−4465)	155 (95−284)	91%, 82%, 76%
**Point‐of‐care RNA testing**	1.8 (1.4−5.6)	4.4 (2.3−17.8)	3305 (1699−9436)	955 (320−3082)	145 (91−275)	93%, 91%, 85%
**No reinfection**						
**Point‐of‐care antibody testing**	2.2 (1.5−2.6)	2.9 (1.6−4.2)	3800 (1697−4626)	640 (211−858)	145 (89−270)	86%, 84%, 79%
**Dried blood spot testing**	2.0 (1.3−2.6)	2.7 (1.4−4.1)	3456 (1533−4521)	585 (184−851)	144 (89−269)	87%, 85%, 81%
**Clinic‐based reflex RNA testing**	1.8 (1.3−2.4)	2.5 (1.3−3.8)	3171 (1467−4131)	535 (176−788)	143 (88−268)	87%, 87%, 82%
**Point‐of‐care RNA testing**	1.5 (0.9−2.3)	2.0 (1.0−3.6)	2507 (1046−4023)	439 (141−665)	141 (87−267)	90%, 89%, 85%
**Higher reinfection rate (1.5** ^*^ **primary infection**)						
**Point‐of‐care antibody testing**	5.7 (2.8−12.2)	15.0 (5.0−43.2)	10,789 (3398−21,751)	3253 (704−8504)	169 (102−303)	86%, 84%, 79%
**Dried blood spot testing**	5.1 (2.4−11.8)	13.7 (4.3−42.0)	9696 (3175−19,842)	2979 (628−8140)	166 (101−301)	87%, 85%, 81%
**Clinic‐based reflex RNA testing**	4.7 (2.3−11.2)	12.7 (4.1−40.6)	8937 (2959−19,274)	2753 (584−8057)	163 (100−295)	87%, 87%, 82%
**Point‐of‐care RNA testing**	3.2 (1.6−8.5)	9.2 (3.1−31.8)	5826 (2152−14,579)	1990 (439−5545)	154 (93−284)	90%, 89%, 85%
**Prioritized MSM who regularly engaged with HIV prevention and care services**						
**Point‐of‐care antibody testing**	4.2 (2.3−7.8)	9.5 (3.5−24.1)	7650 (2719−13,601)	2067 (496−4760)	159 (98−288)	86%, 84%, 79%
**Dried blood spot testing**	3.8 (1.9−7.5)	8.6 (3.1−23.5)	6870 (2504−12,611)	1880 (434−4475)	157 (96−285)	87%, 85%, 81%
**Clinic‐based reflex RNA testing**	3.5 (1.9−7.1)	7.9 (2.9−22.3)	6319 (2374−12,000)	1727 (411−4403)	155 (95−283)	87%, 87%, 82%
**Point‐of‐care RNA testing**	2.5 (1.3−5.5)	5.8 (2.2−17.4)	4295 (1673−9248)	1270 (317−3023)	148 (91−274)	90%, 89%, 85%

*Notes*: Number of people living with chronic HCV, number of annual new infections, number of annual HCV‐related deaths and percentage of diagnosed, treatment initiation and cured rounded to the nearest one. The status‐quo scenario refers to the current HCV testing and treatment practice in Taiwan. Base‐case scenarios refer to scenarios that replace current HCV testing practices with four simplified HCV testing strategies, which change the testing and treatment parameters and other parameters remain as same as the status quo. Best estimate and 95% percentile interval for the 1000 ensemble simulations.

Abbreviation: PY, person‐years.

### 3.1 Impact of simplified HCV testing strategies

**Figure 2:**
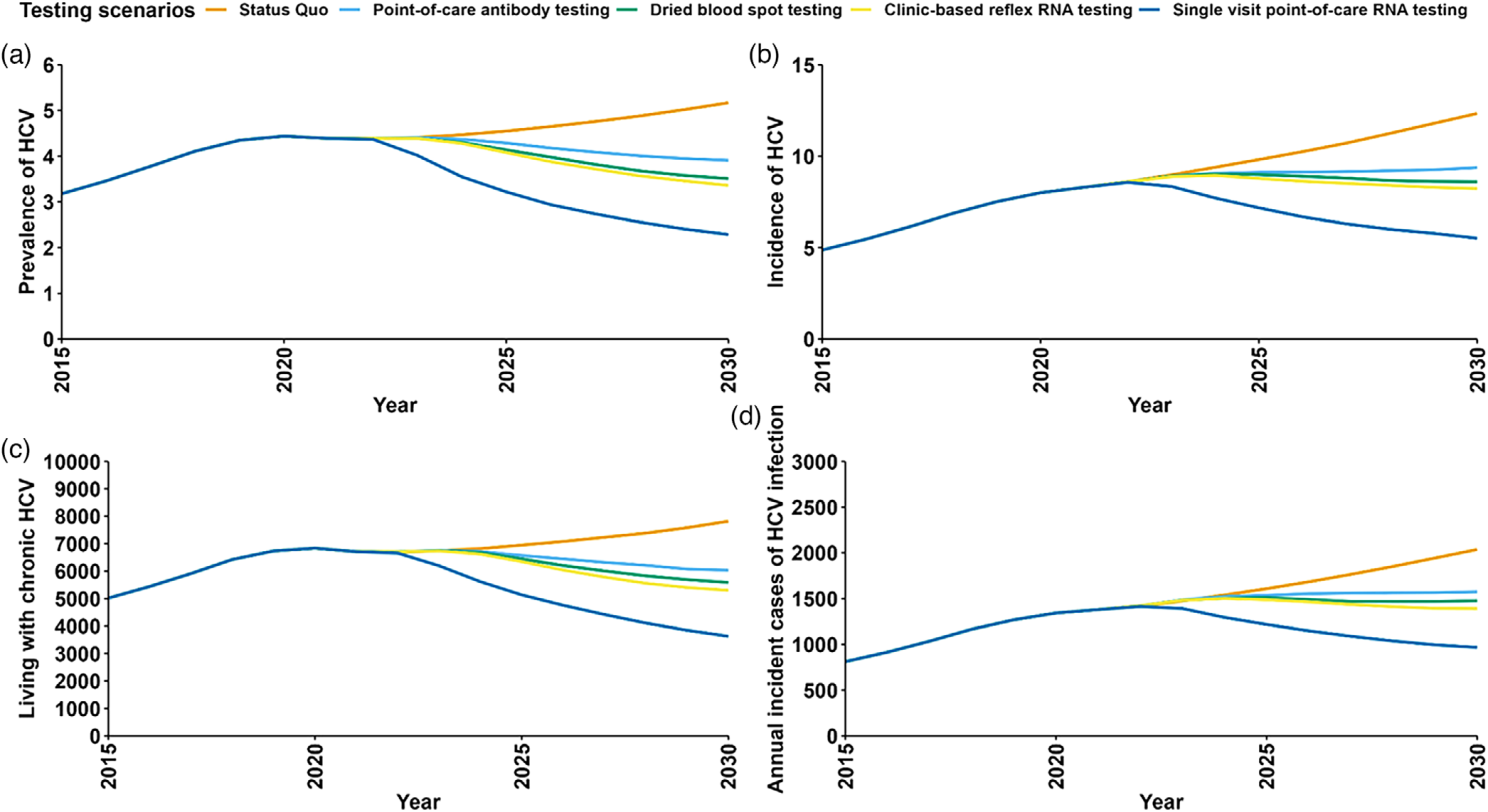
Annual change in **(a)** HCV prevalence, **(b)** HCV incidence, **(c)** number of people living with chronic HCV and **(d)** number of HCV new infections among MSM in Taiwan 2030 (2015−2030, best estimates, percentile intervals shown in Table 2).

**Figure 3:**
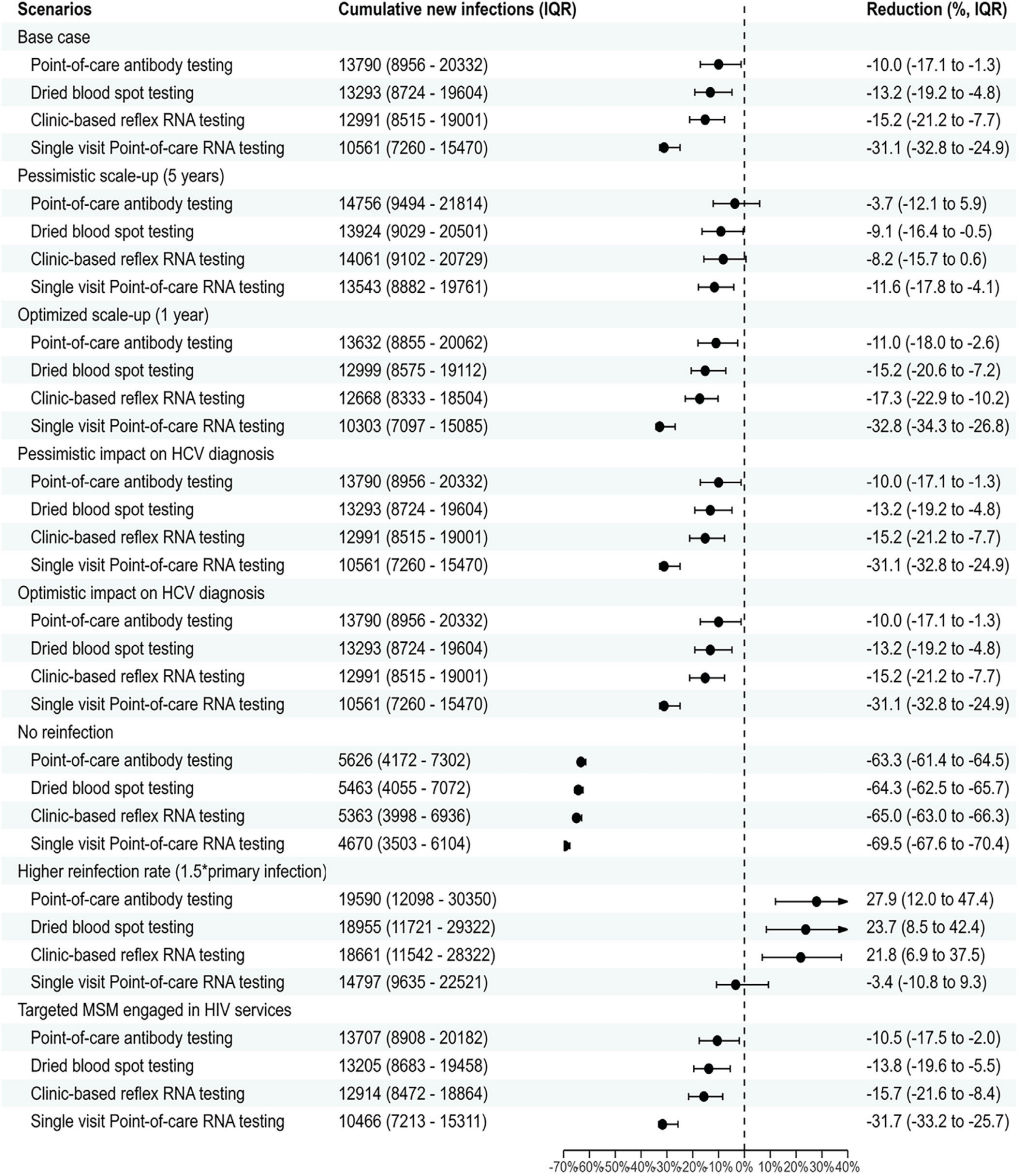
The median (IQR) percentage reduction in new HCV infections over 2022−2030 for all scenarios compared to the status‐quo scenario with the base parameter values. Number of cumulative new infections (IQR) rounded to the nearest one.

After a 2‐year scale‐up, simplified HCV testing strategies could substantially reduce HCV incidence to 5.9−9.6 per 1000 person‐years by 2030 (shown in Figure 2 and Table 2). Among all simplified HCV testing scenarios, the single‐visit point‐of‐care RNA testing scenario leads to the largest reductions in the number of cumulative new HCV infections between 2022 and 2030 compared to the status quo (31.1% reduction, interquartile range [IQR]: 24.9%−32.8%); followed by the clinic‐based reflex RNA testing scenario (15.2% reduction, IQR: 7.7%−21.2%), the DBS testing scenario (13.2% reduction, IQR: 4.8%−19.2%) and the point‐of‐care antibody testing scenario (10.0% reduction, IQR: 1.3%−17.1%), shown in Figure 3.

**Figure 4:**
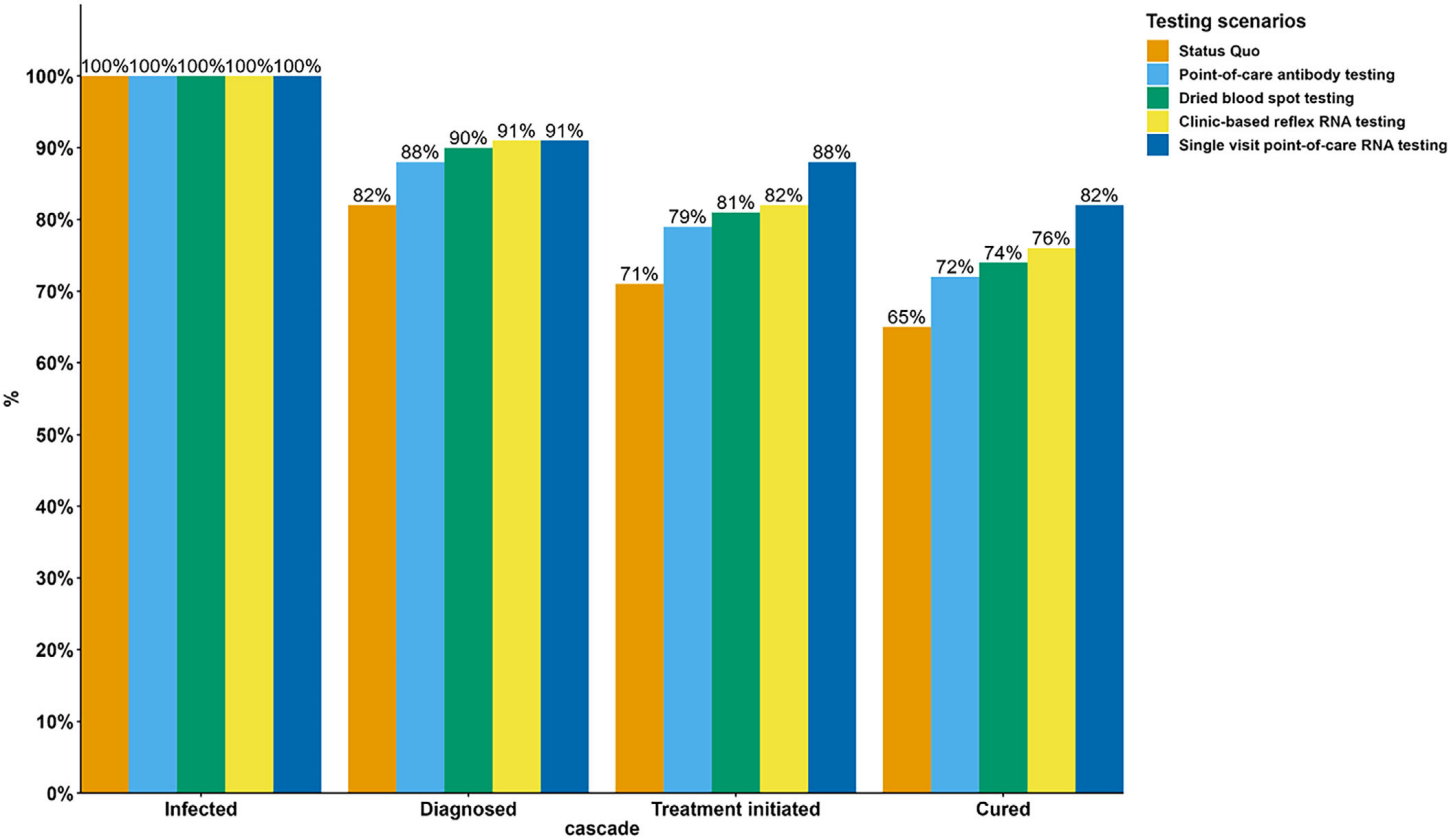
Impact of simplified HCV testing strategies on HCV cascade of care (best estimate percentages for diagnosed, treatment initiation and achieving SVR) among all MSM in Taiwan in 2030.

Additionally, all simplified HCV testing scenarios substantially improve the HCV care cascade by 2030. The percentage of MSM diagnosed with HCV increased to nearly 90% and above (status‐quo scenario: 82%); the percentage of MSM initiated HCV treatment improved to nearly 80% and above (status‐quo scenario: 71%); and the percentage of MSM cured from HCV increased to over 70% (status‐quo scenario: 65%). The single‐visit point‐of‐care RNA testing scenario had the largest improvements in each step of HCV care with 91%, 88% and 82% of MSM living with HCV diagnosed, initiated treatment and cured from HCV, respectively (shown in Figure 4).

The impact of simplified HCV testing strategies on the HCV epidemic demonstrated a similar trend among all MSM subgroups (Figures S8−S12).

### 3.2 Sensitivity analysis

Our sensitivity analysis indicated the reinfection rate had a large impact on the effectiveness of each simplified HCV testing scenario (Table 2). If there was no reinfection among the cured population, HCV incidence would be 65.5%−69.8% lower across simplified HCV testing scenarios than the base case. On the other hand, if the risk of reinfection was 1.5 times higher than primary infection, HCV incidence would increase by 55.9%−58.8% across simplified HCV testing scenarios compared to the base case. Increasing risk of reinfection would offset the impact of simplified testing potentially resulting in an increase in HCV incidence by 2030 compared to the base case (Table 2). Compared to the status quo, prioritizing simplified HCV testing strategies for PrEP users and MSM living with diagnosed HIV had a considerable impact on the HCV epidemic among overall MSM (HCV incidence reduced by 24.6%−54.0% across testing strategies).

Additional results of the sensitivity analysis among the MSM subgroups are shown in Figures S13−S21.

## 4 DISCUSSION

Our modelling examined the current status of the HCV epidemic in Taiwan and assessed the potential impact of different simplified HCV testing strategies on the HCV epidemic among MSM in the era of DAAs. Our model projections show that all simplified HCV testing strategies reduce HCV prevalence and incidence and improve HCV diagnosis and treatment. Among the four simplified HCV testing strategies considered, the single‐visit point‐of‐care RNA testing scenario resulted in the greatest reductions in HCV prevalence and incidence and improved HCV diagnosis and treatment among MSM in Taiwan. Our analysis also suggests that reinfection could mitigate the benefits of simplified HCV testing strategies and, therefore, additional risk reduction strategies may be required. This study provides important data to inform national planning for the scale‐up of simplified HCV testing among MSM in Taiwan. This mathematical model could also be used in other countries globally to evaluate the impact of simplified strategies on HCV testing and treatment.

Few modelling studies have focused on exploring the potential for testing interventions to control HCV epidemics among MSM. Modelling studies in Australia and France focus on the effect of testing and DAA scale‐up among only MSM living with HIV [15, 16]. These studies suggested that increasing HCV screening and additional risk reduction strategies alongside scaling up DAA treatment are crucial to achieve the WHO HCV elimination impact target−an 80% reduction in HCV incidence by 2030 compared to the level of 2015 [32]. However, none of these studies considered the multiple simplified HCV testing strategies in their model, nor did they estimate the impact of testing on the coverage of HCV diagnosis and treatment among the entire population of MSM.

In addition to assessing the impact of simplified HCV testing strategies on HCV incidence and HCV diagnosis and treatment, we further examined the potential for simplified HCV testing strategies to achieve the WHO HCV elimination goals [32]. Our modelling results suggested reflex RNA testing and single‐visit point‐of‐care RNA testing could achieve the WHO hepatitis elimination service coverage target of 80% of those diagnosed with HCV received treatment [32]. Our finding highlights that simplifying the HCV diagnosis and treatment initiation to one visit could substantially improve HCV diagnosis and treatment by preventing loss to follow‐up in the HCV care cascade and increasing HCV treatment uptake. However, none of the simplified HCV testing strategies in our model achieved the WHO elimination impact target for the entire population of MSM (additional results shown in Figure S9A). HCV elimination would be nearly achieved in MSM living with diagnosed HIV who had universal access to and free‐of‐charge DAA treatment and high HCV testing levels while no reinfection occurred. This result may infer that controlling the HCV epidemic among MSM requires a high percentage for the HCV care target, that is diagnosis, treatment initiation and cure, alongside the care cascade as well as additional risk reduction behaviour.

A modelling study among MSM in the UK indicated that scaling up HCV test and treatment targeting PrEP users (25% coverage of PrEP) as well as MSM living with diagnosed HIV could have a significant impact on the HCV epidemic and lead to achieving the WHO HCV elimination goals [12]. Our results in sensitivity analysis suggested considerable impact is possible from just prioritizing simplified HCV testing strategies for PrEP users and MSM living with diagnosed HIV compared to status quo at a low PrEP coverage (1.1%); however, the WHO HCV elimination targets cannot be reached. These results highlight the urgent need for HIV/HCV integrated prevention strategies for achieving HIV/HCV elimination among MSM by 2030.

The study has some limitations. First, model input parameters for the HCV cascade are uncertain with data lacking past and current HCV screening and treatment practices for MSM except for the MSM living with diagnosed HIV in Taiwan. Furthermore, our model incorporates multi‐dimensional parameters to capture the HCV testing and treatment dynamic among MSM. However, this complexity and risk of overparameterization may limit generalizability to future trends. Second, several model assumptions are simplistic due to a lack of represented data for Taiwan. We assumed HCV incidence among HIV negative on PrEP MSM is similar to HIV negative not on PrEP MSM since there is no evidence of increasing HCV incidence among PrEP users in Taiwan. However, in other countries, such as the Netherlands and Belgium, HCV incidence is higher among MSM PrEP users than HIV‐negative MSM [3]. We assumed the reinfection rate equals the primary infection rate due to uncertainties in existing literature, addressing this uncertainty via a sensitivity analysis. Third, we did not include the COVID‐19 pandemic's impact in our projections due to uncertainties regarding its effects on HCV in Taiwan, including potential disruptions to testing, care access and transmission [33]. Further research is needed to investigate its impact on HCV health services. Last, we did not consider the feasibility of implementing different simplified HCV testing strategies, which may impact the real‐world scale‐up and resulting population‐level effectiveness. There is limited HCV point‐of‐care testing currently available in Taiwan for simplifying the HCV diagnosis algorithm; therefore, how well the simplified HCV testing strategies can be integrated into health services in Taiwan and its acceptances by healthcare providers and patients remains unknown. Future work on the real‐world implementation and effectiveness of simplified HCV testing strategies among MSM is urgently needed.

The main strength of our study is that, to our knowledge, it is one of the first modelling studies to examine the impact of various simplified HCV testing strategies on the HCV epidemic as well as HCV diagnosis and treatment among MSM, including those who are HIV negative and accessing PrEP. An advantage of our model is its flexibility in terms of the population structure modelled making it adaptable to other countries and populations for evaluating a range of questions related to HCV simplified testing scale‐up. This study provides useful information for the introduction of simplified HCV testing and its role in controlling the HCV epidemic suggesting that simplified HCV testing approaches should be provided to MSM irrespective of HIV status. Comprehensive interventions, including risk behaviour reduction alongside simplified HCV testing, are likely to be warranted to control HCV among MSM in Taiwan.

## 5 CONCLUSIONS

Our modelling demonstrates the potential for simplified HCV testing strategies to substantially reduce HCV incidence and increase the level of HCV treatment and cure among MSM in Taiwan. Simplified HCV testing strategies could mitigate the increasing HCV burden among this population. Single‐visit point‐of‐care RNA testing has the most impact on the HCV epidemic among MSM. Simplified HCV testing alone will not achieve the WHO HCV elimination targets. The implementation of additional behaviour strategies will be required to pave the way to HCV elimination among MSM in Taiwan.

## COMPETING INTERESTS

RTG has received funding for his research from WHO and has provided non‐funded project advice to Gilead and ViiV. JG is a consultant/advisor and has received research grants from AbbVie, bioLytical, Camurus, Cepheid, Gilead Sciences, Hologic and Indivor, and has received honoraria from AbbVie, Cepheid, Gilead Sciences and Merck. All other authors have no competing interests to declare.

## AUTHORS’ CONTRIBUTIONS

H‐JW, STFS and RTG conceived the study. H‐JW developed the model. H‐JW set up and ran the scenarios. H‐JW designed the analyses and drafted the manuscript. SSTFS, JG and RTG validated the model input. All authors were involved in writing and revising the manuscript.

## FUNDING

This study is made possible as part of a research‐funded PhD being undertaken by HJW under the University of New South Wales (UNSW) Scientia scholarship and associated with the Rapid Point of Care Research Consortium for infectious disease in the Asia Pacific (RAPID), which is funded by an NHMRC Centre for Research Excellence. This study received no funding from pharmaceutical companies or the diagnostics industry. The Kirby Institute is funded by the Australian Government Department of Health and is affiliated with the Faculty of Medicine, UNSW Sydney, Australia.

## DISCLAIMER

The views expressed in this publication do not necessarily represent the position of the Australian Government. JG is supported by a National Health and Medical Research Council Investigator Grant (1176131).

## Supporting information

Supporting Information

## Data Availability

Model parameters are available in tables and Supplementary Materials. The code used to produce the estimates with aggregate data and results for this study are available online at https://github.com/The‐Kirby‐Institute/Simplified‐HCV‐testing‐model. The technical document is available online at the https://github.com/The‐Kirby‐Institute/Simplified‐HCV‐testing‐model/tree/main/02.%20Documents
