# Supplementary material for: Impact of simplified HCV diagnostic strategies on the HCV epidemic among men who have sex with men in the era of HIV oral pre‐exposure prophylaxis in Taiwan: a modelling study
Source: J Int AIDS Soc. 2024 May 2;27(5):e26251. doi: 10.1002/jia2.26251 (PMC11063777; doi:10.1002/jia2.26251)
Supplement: Supplementary file 1 — Supporting Information [file JIA2-27-e26251-s001.docx]

**Impact of simplified HCV diagnostic strategies on the HCV epidemic among men who have sex with men in the era of HIV oral pre-exposure prophylaxis in Taiwan: a modelling study**

**Supplementary material**

Huei-Jiuan Wu^1^ (MSc)*, Sophy TF Shih^1^ (DrPH), Tanya L. Applegate^1^ (PhD), Jisoo A. Kwon^1^ (PhD), Evan B. Cunningham^1^ (PhD), Jason Grebely^1^ (PhD), Richard T. Gray^1^ (PhD)

^1^The Kirby Institute, UNSW, Sydney, Australia

**Contents**

[**1. Model parameterization and calibration** 3](#_Toc157197255)

[**A.** **Population size and HIV epidemics of Taiwanese MSM** 3](#_Toc157197256)

[**I.** **Estimated population size of MSM** 3](#_Toc157197257)

[**II.** **HIV prevalence and incidence among MSM in Taiwan** 3](#_Toc157197258)

[**III.** **Numbers of MSM annually diagnosed with HIV** 3](#_Toc157197259)

[**IV.** **Numbers of MSM living with diagnosed HIV** 4](#_Toc157197260)

[**V.** **Numbers of MSM using PrEP** 4](#_Toc157197261)

[**B.** **HCV prevalence and incidence among entire MSM and each MSM sub-population** 4](#_Toc157197262)

[**C.** **Estimated cumulative number of HCV treatment initiations and people who have achieved SVR among MSM** 4](#_Toc157197263)

[**2. Testing scenarios** 5](#_Toc157197264)

[**3. Additional results** 24](#_Toc157197265)

[**4. Sensitivity analysis** 29](#_Toc157197266)

[**5. References for supplementary material** 40](#_Toc157197267)

# **[1. Model parameterization and calibration](#section1)**

Key parameters are presented in **Table S1**. Parameters were extracted from literature with a preference for studies form Taiwan and from Taiwan HCV policy guideline documents. We assumed the HCV reinfection rates are the same as the primary infection rates, given the uncertainties surrounding reinfection in the existing literature. We address this uncertainty through sensitivity analysis. Detailed model parameters and justifications are provided in Supplementary Material **Table S1**. The uncertainty interval of parameters was obtained either from the literature or, if not available, set to an assumed plausible range. We used available demographic and epidemiological data to calibrate the model to reflect HCV epidemic among Taiwanese MSM over 2004-2020 and future trends up to 2030.

We first used a fixed set of parameters representing the year 2004, running the model to equilibrium following the introduction of 1 person with HCV to obtain the proportional distribution of MSM in each population and HCV infected compartment. The resulting proportions were then used to initialize the population in 2004. From 2004, we allowed the parameters to be time varying. We generated plots to compare the model outputs against observed epidemiological data visually. Iteratively, we manually adjusted key parameters to minimize the discrepancies between model outputs and the epidemiological data. The model calibration process incorporated insights from HCV epidemiology experts and experienced modellers in interpreting visual fit, guiding adjustments, and ensuring that the model's dynamics aligned with epidemiological data. We also visually check the trend of all the transitions and the compartments in the same timeframe to ensure model outputs' accuracy and reliability.

The model intentionally navigates a delicate balance between simplicity and the imperative for a thorough exploration of the HCV epidemic among MSM in the PrEP era. Our primary objective is to assess the impact of simplified HCV testing strategies, prompting us to maintain intricacies within the HCV cascade and population transitions. To mitigate the risk of overparameterization, we make a conscious compromise by simplifying the representation of HCV and HIV transmission dynamics. This strategic decision allows us to focus on the specific complexities inherent in the testing and treatment landscape while maintaining a manageable and interpretable model structure. It simply estimated the number of new infections using a transmission rate per timestep between a susceptible person in the MSM subpopulations and an infected person in the overall modelled population. The force of infection for HCV, $\alpha_{i}$ ,incorporates all the effects of the behaviors contributing to HCV acquisition ($\beta_{i}$) and the level of contact between populations ($\frac{I}{N}$), where N represents the entire modelled population, and I is the number of people living with HCV. In the model it is assumed random interactions occur between sub-populations. Hence,

$\begin{aligned} \alpha_{i} = \frac{\beta_{i}I}{N}.\#\left( 1 \right) \end{aligned}$

We further sampled from the range of each parameter using a uniform distribution to produce 1000 input parameter sets for uncertainty analysis. We reported results using the best estimates and 95% percentile interval from the 1000 model simulation.

As we focused on HCV testing and treatment interventions, the model did not describe HIV transmission directly. Rather HIV-negative MSM acquired HIV at a rate calibrated to match the HIV prevalence and incidence in the population. HIV-negative MSM in the model started and discontinued PrEP from the beginning of 2017 (when it became available in Taiwan). If they acquired HIV they transitioned to HIV-positive undiagnosed and became HIV diagnosed at a fitted diagnosis rate. Given that 92% of HIV-infected individuals who were diagnosed in Taiwan were on HIV antiretroviral therapy (ART),^1^ we assumed that MSM who were HIV diagnosed are on ART.

The indicators used for calibration are as follows:

1. **Population size and HIV epidemics of Taiwanese MSM**

We calibrated the model to the demographics of the entire MSM population in Taiwan. The calibration targets included the estimated population size of MSM, HIV prevalence and incidence among MSM, number of HIV diagnosed MSM, number of MSM living with HIV, proportion of MSM with HIV diagnosed, and number of MSM PrEP users, shown in **Table S2** and **Table S3**. Our calibration efforts focused on key parameters, including the number of PrEP users and MSM living with HIV and diagnosed since data of HIV prevalence and incidence may be subject to selection bias and measurement challenges in the estimating incidence.

1. **Estimated population size of MSM**

The size of the entire MSM population was based on the estimated percentage of men self-identified as non-heterosexual men in Taiwan (1.9%, 95% CI: 1.3% -2.5%). This estimate is similar to the estimates from other high income countries.^3–5^

1. **HIV prevalence and incidence among MSM in Taiwan**

HIV prevalence and incidence among MSM in Taiwan were obtained from published literature. HIV prevalence data was extracted from three studies conducted in Taiwan.^6–8^ We review the representativeness of MSM populations in the studies and had a preference for data collected from multiple centers since there was not a single cohort study reporting the HIV prevalence over 2004 to 2020. HIV incidence data and the proportion of MSM living with HIV diagnosed were also extracted from different sources.^1,6,8^ While these data represent the best available, they may be subject to selection bias and measurement challenges in estimating incidence.

1. **Numbers of MSM annually diagnosed with HIV**

The annual number of MSM diagnosed with HIV was assumed to equal the number of notifications each year extracted from Taiwan Centers for Disease Control (Taiwan CDC) notification database.^9^ The annual number of MSM HIV diagnosed cases was assumed to equal the sum of notifications for males attributing exposure to homosexual contact HIV diagnosed cases and unknown exposure HIV diagnosed cases. We estimated the number of MSM HIV cases in this way to mitigate the bias, which contributed to homosexual-related stigma, on self-report HIV exposure in the notification database. The upper bound of estimated annual number of MSM newly diagnosed with HIV was calculated as sum of notifications for all males attributing exposure to non-injecting drugs HIV diagnosed cases. The lower bound of estimated annual number of MSM newly diagnosed with HIV was calculated as sum of notifications for all males attributing exposure to homosexual contact HIV diagnosed cases.

1. **Numbers of MSM living with diagnosed HIV**

The number of MSM living with diagnosed HIV infection was the cumulative sum of the number of notifications, excluding the cumulative number of deaths among MSM diagnosed with HIV. The data source was also extracted from the Taiwan Centers for Disease Control (Taiwan CDC) notification database.^9^ The estimated method for this indicator was as same as for the number of MSM annually diagnosed with HIV.

1. **Numbers of MSM using PrEP**

We also calibrated the model to the number of MSM PrEP users. PrEP has been available in Taiwan since the end of 2016. MSM who are HIV-negative not on PrEP starting PrEP and MSM who are HIV-negative on PrEP stopping PrEP at rates calibrated to match estimated number of MSM PrEP users in Taiwan. The number of MSM PrEP users was extracted from literatures.^10,11^

1. **HCV prevalence and incidence among entire MSM and each MSM sub-population**

There are no data on HCV prevalence and incidence among all MSM subpopulations in our model. We assumed HCV prevalence and incidence are the same for HIV-negative and HIV-positive MSM since HCV and HIV share similar transmission routes. Although studies suggest PrEP for HIV among MSM may associated with viral transmission of HCV through high-risk sexual behaviors and high HCV incidence observed among MSM PrEP users from other Western European countries, there is no data on HCV incidence and prevalence on MSM who are on PrEP in Taiwan. We therefore assumed the HCV incidence and prevalence are the same for the HIV-negative sub-populations regardless of PrEP use.

1. **Estimated cumulative number of HCV treatment initiations and people who have achieved SVR among MSM**

There are data for cumulative number of HCV treatment initiations and the number of people who have achieved SVR in the general population of Taiwan.^12^ We assumed there was no HCV treatment before 2003. There were around 95000 people received HCV treatment under Taiwan National Health Insurance (NHI)between 2003 to 2017 prior to availability of DAAs in Taiwan, corresponding to 20% to 30% treatment coverage by 2017.^12^ However, the NHI data did not provide the characteristics of HCV patients. We therefor assumed the cumulative number of HCV treatment initiations among all MSM as 20% to 30% of the cumulative number of HCV cases in 2017. The combination of pegylation or addition of polyethylene glycol (PEG) to interferon (IFN) and ribavirin (PEG–IFN/ribavirin) for 24 weeks has been the standard of care for people living with HCV since 2004 until the arrival of DAAs in Taiwan. The rate of SVR for PEG–IFN/ribavirin 24 weeks was 72% among people living with HIV and HCV coinfection.^13^ We estimated the number of MSM living with HCV achieved SVR by the HCV treatment coverage among overall MSM multiplied by the rate of SVR for PEG–IFN/ribavirin 24 weeks.

**Figure S2** to **Figure S*7*** show the model projections compared to available epidemiological data. The figures show a best estimates simulation and 95% percentile interval from the 1000 model simulations to reflect the uncertainty in the input parameters.

# **2. Testing scenarios**

These scenarios are described as follows:

(1) Status quo (reference scenario): current practice of HCV testing and treatment in Taiwan, that is, laboratory-based HCV antibody testing for detection of antibodies of HCV at the first visit followed by confirmation of current HCV infection by laboratory-based HCV RNA testing at the second visit, then further access to HCV treatment at the third visit (three clinical visits for HCV treatment initiation).

(2) Point-of-care HCV antibody testing: In this scenario, people receive point-of-care HCV antibody testing with fingerstick blood samples and obtain the testing results within 30 minutes in nonclinical settings, such as outreach sites. Those diagnosed positive require a separate visit for HCV RNA testing and access to HCV treatment if confirmation of current HCV infection at a third visit. We assumed point-of-care HCV antibody testing only increased coverage of people who received HCV antibody testing due to a separate visit required for HCV RNA testing. Although three visits are required for HCV treatment initiation in this scenario, the finger-stick capillary whole-blood testing modelled could expand HCV screening into nonclinical settings since it does not require a phlebotomy blood sample.

(3) Dried blood spot (DBS) testing: We modelled a DBS testing strategy based on fingerstick blood samples collected on paper cards and performed in clinics by trained staff. We assumed samples would be processed in central laboratories for HCV antibody testing and RNA testing conducted if the antibody was positive (reflex RNA testing). Despite evidence that DBS improves the uptake of HCV antibody testing and RNA testing, we assumed HCV treatment initiation in the DBS scenario would be the same level as the status quo due to the requirement of central laboratory testing (two visits for HCV treatment initiation).

(4) Clinic-based reflex RNA testing: We modelled a reflex RNA testing strategy where an initial onsite antibody point-of-care test returning a positive result would be followed by a laboratory-based HCV RNA test in the same visit and access to HCV treatment in a second visit if current HCV infection is confirmed. Blood samples obtained at the time of collection initiation for HCV antibody testing and subsequent HCV RNA if the HCV antibody test is confirmed positive thus promote linkage to HCV care. People receiving clinic-based reflex RNA testing could access HCV treatment within two visits.

(5) Point-of-care RNA testing: People were tested with a point-of-care HCV RNA test onsite only and provided treatment onsite following a positive result. In this scenario, we assumed point-of-care HCV RNA testing replaces current antibody testing and an HCV antibody diagnosis was not required (single visit for diagnosis and treatment initiation).

**[Table S1.](#TableS1) Model parameterization. The symbols representing each parameter in the equations are accompanied by their respective values, expressed as percentages per annum (point estimates). A plausible range is set by the point estimates ± 25% where “NA” is provided in the range column. Liver fibrosis stages, categorized based on the METAVIR scoring system from F0 to F4 (F0—no fibrosis, F1—portal fibrosis, F2—periportal fibrosis, F3—bridging fibrosis, F4—cirrhosis), are indicated. Abbreviations for other liver conditions include DC for decompensated cirrhosis, HCC for hepatocellular carcinoma, LF for liver failure requiring liver transplant, PLF for liver failure followed by liver transplant, and SVR for Sustained Virologic Response.**

| **Parameters (symbol)** | **Value (percentage per annum)** | | **Range** | **Reference** | **Footnote** |
| --- | --- | --- | --- | --- | --- |
| **Demographics** | | | | | |
| Size of MSM population ($N$) | 1.9%* male population | | NA | ^14,15^ | 1 |
| Background mortality ($\mu_{1}, \mu_{2}$) | 0.9% | | NA | ^14^ | 2 |
| Mortality rate among HIV-positive undiagnosed population ($\mu_{3}$) | 1.0% | | NA | ^16^ | 3 |
| Mortality rate among HIV-positive diagnosed population ($\mu_{4}$) | 0.9% | | NA | ^16^ | 3 |
| **Population transition** | | | | | |
| HIV negative → HIV negative on PrEP ($p_{1,2}$) | 0.4% | | NA |  |  |
| HIV negative on PrEP→HIV negative ($p_{2,1}$) | 11.0% | | NA |  |  |
| HIV negative →HIV-positive undiagnosed ($p_{1,3}$) | 1.1% | | NA |  |  |
| HIV negative on PrEP →HIV-positive undiagnosed ($p_{2,3}$) | 0.1% | | NA |  |  |
| HIV-positive undiagnosed →HIV-positive diagnosed ($p_{3,4}$) | 28.0% | | NA |  |  |
| **HCV natural history** |  |  |  |  |  |
| Transmission probability ($\beta_{i}$) | HIV negative | 7.7% | NA |  | 4 |
|  | HIV negative and on PrEP | 2004-2017:0.0%  2017-: 7.7% | NA |  |  |
|  | HIV positive undiagnosed | 66.7% | NA |  |  |
|  | HIV positive diagnosed | 63.2% | NA |  |  |
| Spontaneous clearance probability (${sp}_{i}$) | HIV positive | 15.4% | 11.5%-19.3% | ^17^ |  |
|  | HIV negative | 26.0% | 22.0%-29.0% | ^18^ |  |
| Acute → F0 ($\sigma_{AF0, i}$) | HIV positive | 86.5% | 73.6%-98.2% | ^19^ | 5 |
|  | HIV negative | 86.5% | 73.6%-98.2% | ^19^ |  |
| F0 → F1 ($\sigma_{F0F1, i}$) | HIV positive | 12.2% | 9.8%–15.3% | ^20^ |  |
|  | HIV negative | 11.7% | 10.4%-13.0% | ^21^ |  |
| F1→ F2 ($\sigma_{F1F2, i}$) | HIV positive | 11.5% | 9.5%–14.0% | ^20^ |  |
|  | HIV negative | 8.5% | 7.5%-9.6% | ^21^ |  |
| F2→ F3 ($\sigma_{F2F3, i}$) | HIV positive | 12.4% | 9.7%–15.9% | ^20^ |  |
|  | HIV negative | 12.0% | 10.9%–13.3% | ^21^ |  |
| F3→ F4 ($\sigma_{F3F4, i}$) | HIV positive | 11.5% | 9.8%–13.5% | ^20^ |  |
|  | HIV negative | 11.6% | 10.4%–12.9% | ^21^ |  |
| F3→ HCC ($\sigma_{F3HCC, i}$) | HIV positive diagnosed | 2. 9% | 1.2%-5.6% |  | 6 |
|  | HIV positive undiagnosed | 4.3% | 1.8%-8.3% |  | 6 |
|  | HIV negative | 1.7% | 0.7%–3.3% | ^22^ |  |
| F4→ DC ($\sigma_{F4DC, i}$) | HIV positive | 4.6% | 2.9%–6.3% | ^23^ | 7 |
|  | HIV negative | 4.6% | 2.9%–6.3% | Assumption | 7 |
| F4→ HCC ($\sigma_{F4HCC, i}$) | HIV positive diagnosed | 7.3% | 2.4% - 14.1% |  | 6 |
|  | HIV positive undiagnosed | 10.8% | 3.5% - 20.8 % |  | 6 |
|  | HIV negative | 4.3% | 1.4%-8.3% | ^24^ |  |
| DC→ HCC ($\sigma_{DCHCC, i}$) | HIV positive diagnosed | 11.6% | 7.0%–16.8% |  | 6 |
|  | HIV positive undiagnosed | 17.0% | 10.3%–24.8% |  | 6 |
|  | HIV negative | 6.8% | 4.1%–9.9% | ^25^ |  |
| DC→ liver failure requiring liver transplant (LF) ($\sigma_{DCLF, i}$) | HIV positive diagnosed | 5.6% | 2.9%-8.3% |  | 6 |
|  | HIV positive undiagnosed | 8.3% | 4.3%-12.3% |  | 6 |
|  | HIV negative | 3.3% | 1.7%-4.9% | ^26^ |  |
| HCC→ LF ($\sigma_{HCCLF, i}$) | HIV positive diagnosed | 17.0% | 8.5%-30.6% |  | 6 |
|  | HIV positive undiagnosed | 25.0% | 12.5%-45.0% |  | 6 |
|  | HIV negative | 10.0% | 5.0%-18.0% | ^27^ |  |
| LF→PLT ($\sigma_{LFPLF, i}$) | HIV positive | 0 | NA | Assumption | 8 |
|  | HIV negative | 0 | NA | Assumption | 8 |
| DC-related death (${\mu DC}_{i}$) | HIV positive | 40.54% | 27.09%-60.64% | ^28^ | 9 |
|  | HIV negative | 26.0% | NA | ^28^ |  |
| HCC-related death (${\mu HCC}_{i}$) | HIV positive | 47.9% | 42.7%-54.5% | ^29^ | 10 |
|  | HIV negative | 17.7% | 15.8%-20.2% | ^30^ |  |
| LF-related death (${\mu LF}_{i}$) | 58.3% | | NA | ^31^ | 11 |
| PLF-related death (${\mu PLF}_{i}$) | 0.0% | |  | Assumption | 8 |
| F3→ HCC (post cure) (${\sigma c}_{F3HCC,i}$) | 0.2% | | 0.1%-0.6% | Assumption | 12 |
| F3→ f4 (post cure) (${\sigma c}_{F3F4,i}$) | 0.2% | | 0.1%-0.6% | Assumption | 12 |
| F4→ DC (post cure) (${\sigma c}_{F4DC,i}$) | 0.2% | | 0.1%-0.6% | Assumption | 12 |
| F4→ HCC (post cure) (${\sigma c}_{F4HCC,i}$) | 0.2% | | 0.1%-0.6% | ^32^ |  |
| DC→ HCC (post cure) (${\sigma c}_{DCHCC,i}$) | 2.0% | | 0.5%-4.3% | ^33^ |  |
| DC→ LF(post cure) (${\sigma c}_{DCLF,i}$) | HIV positive diagnosed | 5.6% | 2. 9%-8. 3% | Assumption | 13 |
|  | HIV positive undiagnosed | 8.3% | 4.3%-12.3% | Assumption | 13 |
|  | HIV negative | 3.3% | 1.7%-4.9% | Assumption | 13 |
| HCC→ LF (post cure) (${\sigma c}_{HCCLF,i}$) | HIV positive diagnosed | 17.0% | 8.5%-30.6% | Assumption | 13 |
|  | HIV positive undiagnosed | 25.0% | 12.5%-45.0% | Assumption | 13 |
|  | HIV negative | 10.0% | 5.0%-18.0% | Assumption | 13 |
| LF → PLF (post-cured) (${\sigma c}_{LFPLF,i}$) | HIV negative | 0.0% | NA |  |  |
|  | HIV positive | 0.0% | NA |  |  |
| DC-related death (post-cured) (${\mu DC_{0}}_{i}$) | HIV negative | 17.9% | 13.0%-24.7% | ^34^ | 14 |
|  | HIV positive | 41.7% | 27.9%-62.4% | ^34^ | 14 |
| HCC related death (post-cured) (${\mu HCC_{0}}_{i}$) | HIV negative | 6.0% | 2.3%-15.6% | ^35^ | 15 |
|  | HIV positive | 16.3% | 14.5%-18.5% | ^35^ | 15 |
| **HCV care cascade** | | | | | |
| **IFN era 2003-2017** | | | | | |
| HCV antibody testing rate ($\tau_{ab, i}^{X}$) | HIV negative | 10.0% | 6.2%-15.4% | ^36^ | 16 |
|  | HIV negative and on PrEP | 10.0% | 6.2%-15.4% |  | 16 |
|  | HIV positive undiagnosed | 10.0% | 6.2%-15.4% |  | 16 |
|  | HIV positive diagnosed | 63.2% | 48.7%-86.5% | ^37^ | 17 |
| HCV RNA testing rate ($\tau_{RNA, i}^{X}$) | HIV negative | 50.0% | 37.5%-62.5% | ^38^ |  |
|  | HIV negative and on PrEP | 50.0% | 37.5%-62.5% | ^38^ |  |
|  | HIV positive undiagnosed | 50.0% | 37.5%-62.5% | ^38^ |  |
|  | HIV positive diagnosed | 50.0% | 37.5%-62.5% | ^38^ |  |
| Treatment uptake rate ($\eta_{i}^{X}$) | HIV negative | 13.7% | NA | ^39^ |  |
|  | HIV negative and on PrEP | 13.7% | NA | ^39^ |  |
|  | HIV positive undiagnosed | 13.7% | NA | ^39^ |  |
|  | HIV positive diagnosed | 34.4% | NA | ^2^ | 18 |
| SVR rate ($\gamma_{i}^{X}$) | 70.2% | | NA | ^40^ | 22 |
| Treatment failed ($\psi_{i}^{X}$) | 29.8% | | NA | ^40^ | 19 |
| Re-treatment ($\phi_{i}^{X}$) | 4.2% | | NA | ^41^ |  |
| **DAA era 2017 - 2021** | | | | | |
| HCV antibody testing rate ($\tau_{ab, i}^{X}$) | HIV negative | 25.0% | 18.8%-31.3% | ^36^ | 20 |
|  | HIV negative and on PrEP | 25.0% | 18.8%-31.3% | ^36^ | 20 |
|  | HIV positive undiagnosed | 25.0% | 18.8%-31.3% | ^36^ | 20 |
|  | HIV positive diagnosed | 80.0% | 60.0%-98.0% | Assumption | 20 |
| HCV RNA testing rate ($\tau_{RNA, i}^{X}$) | HIV negative | 50.0% | 45.0%-57.0% | ^38^ |  |
|  | HIV negative and on PrEP | 50.0% | 37.5%-62.5% | ^38^ |  |
|  | HIV positive undiagnosed | 50.0% | 37.5%-62.5% | ^38^ |  |
|  | HIV positive diagnosed | 80.0% | 60.0%-98.0% | Assumption | 21 |
| Treatment uptake rate ($\eta_{i}^{X}$) | HIV negative | 27.0% | 20.2%-33.7% | ^2^ | 18 |
|  | HIV negative and on PrEP | 27.0% | 20.2%-33.7% | ^2^ | 18 |
|  | HIV positive undiagnosed | 27.0% | 20.2%-33.7% | ^2^ | 18 |
|  | HIV positive diagnosed | 67.8% | 50.9%-84.8% | ^2^ | 18 |
| SVR rate ($\gamma_{i}^{X}$) | 91.9% | | NA | ^42^ | 23 |
| Treatment failed ($\psi_{i}^{X}$) | 9.1% | | NA | ^42^ |  |
| Reinitiated treatment due to treatment failed ($\phi_{i}^{X}$) | 10% | | NA | Assumption | 24 |

Footnotes:

1. Around the world, the most common estimates of the non-heterosexual population aged 16 years or older range from 1% to 10% in high-income countries.^3–5^ We extracted the estimate of 1.9% (95% CI:1.3% -2.5%) from a study estimating the size of the Taiwan MSM population in 2012.^15^ This was then applied to the estimated population of males aged 15 to 74 to produce the population in the model.
2. The background mortality rate was assumed to equal the mortality rate of the Taiwanese male population aged 15-74 in 2004.^14^ We assume the background mortality rate remained fixed.
3. A previous study on life expectancy of HIV-infected Taiwanese MSM suggested a loss in life expectancy of 6.71±0.06 years for the early diagnosed HIV-infected MSM versus 13.76±0.05 years for those diagnosed late when compared to age- and sex-matched reference population (late diagnosed was defined as the presence of AIDS within one month of HIV diagnosis).^16^ We calculated the mortality rate as the 1/life expectancy. To use the parameters in our difference equation model, we converted this rate to an annual probability using the formula $1-e^{-rate}$.
4. A previous modelling study of HCV among MSM suggested that transmission probabilities for initial HCV infection and subsequent reinfection post cure are equal.^43^ The data on reinfection in era of DAAs is limited and remains controversial between studies among MSM. For this reason, we assume that transmission probabilities are equal for both initial HCV infection and subsequent reinfections.
5. Most studies used the time to spontaneous clearance of HCV as the time from acute stage to F0 (for those who did not spontaneous clear, their HCV progressed from acute to F0 stage over the same time frame). The definition of chronic HCV infection is that the HCV virus did not spontaneous cleared after 6 months.^19^ The estimated is 6 months (3-9 months) referred to a rate of 2/year (1.3/year - 4/year). The yearly probability of progressed from acute to F0 is then $1-e^{-\mu t}$= $1-e^{-2}$=0.86.
6. We were unable to find specific estimates for the disease progression rate among HIV-infected MSM in published literature. A Previous study suggested that stage-specific fibrosis progression rate for chronic hepatitis C virus infection was different for patients on highly active antiretroviral therapy (HAART) for HIV infection.^20^ The risk ratio (RR) was 2.5 (95%CI: 1.8-3.4) and 1.7 (95%CI: 1.1-2.8) for non-HAART group and HAART group, respectively. We assumed disease progression rates (F3→ HCC, F4→ HCC, DC→ HCC, DC→ LF and HCC→ LF) among HIV-undiagnosed and HIV diagnosed MSM on HIV treatment had the same risk ratio and disease progression rates as published in the study.
7. The referenced study included 340 HIV–HCV co-infected patients with compensated (n = 248) or decompensated (n = 92) cirrhosis. The incidence rate of first hepatic decompensation in patients with compensated cirrhosis was 4.62 per 100 persons-years (95% CI 2.91–6.33).^23^ To our best knowledge, there is limited knowledge on the risk of developing compensated cirrhosis among HCV mono-infected MSM. Hence, we assumed the progression rate from F4 to hepatic decompensation among HCV mono-infected individuals was as same as the rate among HIV/HCV co-infected individuals.
8. The referenced study suggested a low prevalence of liver transplantation in Taiwan.^44^ Only 1.45 to 16.63 per million Taiwanese males received a liver transplant over 2000-2013. Most HCV patients who need a liver transplant have died before transplantation in Taiwan (personal communication). Hence, we assumed the rate of liver transplant equals zero regardless of HIV status.
9. A study suggested that HIV/HCV coinfection shortens the life of HCV patients in DC stage.^28^ The 1-year survival estimates were 74% among participants without HIV infection which equates to an annual probability of death of 26% (100% - 74%) for HCV mono infected individuals with DC. We estimated the annual probability of death among HCV/HIV coinfected individuals using the relative risk of death for HIV-infected patients (RR: 2.26, 95% CI: 1.51-3.38).^28^
10. A study conducted in a male prison in the United States between 2003 and 2007 suggested HIV/HCV infection resulted in elevated rates of HCC mortality with an odds ratio of 2.7 (95% CI: 1.3-5.8). We used this odd ratio to estimate the HCC-related death among the HIV-positive population compared to the HCV mono-infected population (2.7*17.73%, 95% CI: 2.7*15.82%-2.7*20.19%).
11. A study suggested that 40.02% of patients with HCV related acute-on-chronic liver failure died within 90 days. We converted this probability to an annual mortality as follows 1-(1-0.402)^(365/90)=0.87572. For use in our difference equation model, we converted it to annual probability as $1-e^{-0.87572}$(58.34%).
12. Most HCV patients with sustained viral suppression rarely progress to next stage (personal communication with local HCV expert). A study estimated the incidence rate of developing HCC among patients in stage F4 with SVR to be 0.23 (95% CI: 0.08–0.62)/100PY.^32^ Hence, we assumed liver individuals who were cured before F3 stage would stay at the same liver fibrosis stage after cure. We were unable to find specific estimates for the disease progression rate among people cured from HCV in other advanced liver fibrosis stages (F3 to HCC, F3 to F4, F4 to DC). We assumed people transitioning from F3 to HCC, F3 to F4, F4 to DC have same transition rate as people transitioning from F4 to HCC stage among HCV SVR patients.
13. We assumed progression from decompensated cirrhosis and HCC to liver failure was not impacted by HCV status since liver failure requiring liver transplant occurs at a very late stage of liver disease.
14. One study showed that liver-related mortality risk was significantly reduced among patients diagnosed with decompensated cirrhosis in the DAA era (November 2014–December 2018) compared with the pre-DAA era (2005–October 2014), with an adjusted hazard ratio: 0.69 (95% CI:0.50–0.95).^34^ We applied this hazard ratio to estimate the liver-related death for people in the cured DC stage in our model.
15. A study estimated increased survival in patients with HCV-related HCC between those cured and a non-treatment group. It showed a 66% reduction in risk of 5-year liver-related mortality in those who achieved SVR (HR: 0.34, 95% CI: 0.13-0.88). In the model we used a point estimate of 6.03% (17.73%*0.34) with 95% CI: 2.30%-15.60%.
16. We found no data on HCV testing coverage among MSM in Taiwan between 2003-2017 (era of IFN-based HCV treatment). One study reported that 30.8% of MSM received HIV testing once in a year.^36^ We assumed the coverage of HCV testing was 20% to 50% of the HIV testing coverage among HIV-negative and HIV undiagnosed MSM. Given a HIV testing coverage of 30.8% testing was 10% with range from 6.16% to 15.4%.
17. In Taiwan, people living with HIV are advised to test for HCV annually. We assumed that MSM living with diagnosed HIV and on treatment will return to clinic for HCV screening every 12 months, which equates to an HCV testing rate of once per year and corresponds to an annual probability of 63.21% in our difference equation model.
18. Most studies reporting HCV treatment initiation among MSM were conducted in Europe (Swiss HIV cohort, ATHENA observational cohort in Netherlands and study in Canada).^2,45,46^ In the ATHENA study in the Netherlands, treatment uptake in the previous 12 months among MSM living with HIV was 34.4% for Interferon (IFN) and then increased to 67.8% once DAA treatment became available.^2^ We assumed similar treatment initiation rates in our study because Taiwan specific data was from single centre study, which may have limited generalisability to all MSM living with HIV in Taiwan.^47^ For the other sub-populations we used the same relative increase in HCV treatment initiation of 1.97 from the IFN era to the DAA era as reported in the ATHENA study. Therefore, the annual probability of treatment initiation in the DAA era for HIV negative and HIV undiagnosed MSM was estimated to be 26.99% (13.7%*1.97).
19. Treatment failure was defined as people who received HCV treatment but did not achieve SVR. Hence, the treatment failure rate equals 1 minus the annual probability of SVR.
20. To our best knowledge, no study has reported HCV testing frequency among MSM in Taiwan in the era of DAAs. However, HCV screening has been incorporated into HIV anonymous testing in recent years.^36,48,49^ We assumed that the proportion of HCV screening incorporated into HIV testing service increased to 75% by 2021. A study suggested that 33% of people at high risk of HIV infection regularly received HIV testing in 2019. Hence, we estimated HCV testing increased from 10% in 2017 to 25% (33%*75%) in 2021 and remained at 25% afterward. We assumed HCV testing increased among HIV-diagnosed MSM from receiving HCV testing every year to every 7 months.
21. Infectious disease physicians in Taiwan have been able to prescribe DAA treatments to HIV/HCV coinfected patients since 2017. This is a patient-centred approach and has the potential to reduce the lost-to-follow-up due to referral. We therefore assumed HIV-diagnosed MSM have a higher probability to receive HCV RNA testing in the DAA era.
22. In Taiwan, the HCV genotype 2a is the dominated genotype among MSM living with HIV, followed by 3a and 6a which have become more common in recent years.^50^ Among MSM living with HIV and HCV, the genotype was HCV- 1/6 in 48.6% of people with the remaining 51.4% having genotype HCV-2/3. The reported SVR rate was approximately 74% to 77% for HCV-1 with 48 weeks of PegIFN/RBV and 86% to 95% for HCV-non-1 with 24 weeks of PegIFN/RBV in Taiwan.^51–53^ A study conducted among MSM living with HIV suggested that the SVR rates for IFN-based treatments were 68% and 72% in patients with chronic HCV-1/6 and HCV-2/3 infection.^40^ We uses a proportional average to produce an overall SVR rate using the HCV genotype distribution in MSM living in HIV in Taiwan. This results in an SVR rate of 70.06%.
23. The data was extract from the clinical trials. Taiwan's NHS database showed that 91.9% of HCV patients who receive DAA reached the SVR in 2017.^42^
24. We assumed that 10% of people who experienced HCV treatment failure would reinitiate HCV treatment every year. This assumption is based on Australian data (personal communication) and the higher acceptability of DAAs than IFN-based regimens.

**[Table S2.](#TableS2) Estimated MSM population between 2004-2020 in Taiwan.**

| **Year** | **Estimated number of 15-year-old MSM entering the overall MSM population each year (95% CI)** | **Estimated overall MSM population size (95% CI)** |
| --- | --- | --- |
| 2004 | 3,063 (2,096-4,030) | 175,802 (120,286-231,319) |
| 2005 | 3,326 (2,276-4,377) | 177,484 (121,437-233,532) |
| 2006 | 3,166 (2,166-4,166) | 179,180 (122,597-235,763) |
| 2007 | 3,176 (2,173-4,179) | 180,648 (123,601-237,695) |
| 2008 | 3,212 (2,198-4,226) | 182,205 (124,667-239,744) |
| 2009 | 3,183 (2,178-4,188) | 183,662 (125,664-241,661) |
| 2010 | 3,191 (2,183-4,199) | 185,135 (126,671-243,598) |
| 2011 | 3,184 (2,178-4,189) | 186,552 (127,641-245,463) |
| 2012 | 3,196 (2,187-4,205) | 187,982 (128,619-247,345) |
| 2013 | 2,641 (1,807-3,475) | 188,852 (129,214-248,489) |
| 2014 | 2,804 (1,919-3,690) | 189,814 (129,873-249,755) |
| 2015 | 3,034 (2,076-3,992) | 190,980 (130,670-251,289) |
| 2016 | 2,541 (1,738-3,343) | 191,581 (131,082-252,080) |
| 2017 | 2,445 (1,673-3,217) | 192,102 (131,438-252,766) |
| 2018 | 2,263 (1,548-2,977) | 192,436 (131,666-253,205) |
| 2019 | 2,177 (1,490-2,865) | 192,686 (131,838-253,535) |
| 2020 | 2,057 (1,407-2,706) | 192,559 (131,751-253,368) |

The population size of MSM was estimated by multiplying the overall adult male population size (aged 15 and above) in Taiwan multiplied by the estimated proportion of males that are MSM. The proportion of the male population in Taiwan estimated to be MSM is 1.9% (95% CI: 1.3%- 2.5%)^15^. The male population aged 15 and above was extracted from publicly available data provided by the Department of Household Registration Affair, Ministry of the Interior, Taiwan.^14^

**[Table S3.](#TableS3) HIV prevalence, incidence, diagnosed number, and proportion of HIV diagnosis among MSM.**

| **Year** | **Number of annual HIV diagnosed MSM** | **Number of MSM living with HIV (diagnosed)** | **HIV prevalence** | **HIV incidence** | **Proportion of HIV diagnosed** | **Number of MSM PrEP users** |
| --- | --- | --- | --- | --- | --- | --- |
| 2004 | 805 | 2897 | 3.43% |  |  |  |
| 2005 | 811 | 3436 | 3.11% |  |  |  |
| 2006 | 941 | 4003 | 4.11% |  |  |  |
| 2007 | 1049 | 4717 | 2.84% |  |  |  |
| 2008 | 1262 | 5559 | 3.46% |  |  |  |
| 2009 | 1368 | 6572 |  |  |  |  |
| 2010 | 1580 | 7713 |  |  |  |  |
| 2011 | 1747 | 9050 |  |  |  |  |
| 2012 | 2023 | 10578 | 4.38% | 3.29 per 100 PY* | 71% |  |
| 2013 | 1903 | 12400 | 6.56% | 5.97 per 100 PY* |  |  |
| 2014 | 1915 | 14171 | 4.53% | 3.97 per 100 PY* | 75% |  |
| 2015 | 1962 | 15978 | 1.84% | 2.08 per 100 PY* |  |  |
| 2016 | 2068 |  |  |  | 79% |  |
| 2017 | 2166 |  |  |  |  | 282 |
| 2018 | 1705 |  |  |  | 84% | 756 |
| 2019 | 1671 |  |  |  | 86.4% | 1150 |
| 2020 | 1135 |  |  |  |  | 1610 |

*PY: person-years.

**[Figure S1.](#FigureS1) Model schematic. (**A) HCV transmission and disease progression. Dashed arrows represent mortality, S: susceptible and never infected with HCV, A: acute HCV infection, F0-F4: chronic HCV infection classified by stage of liver fibrosis with METAVIR score, DC: decompensated cirrhosis, HCC: hepatocellular carcinoma, LF: liver failure and requires treatment, PLF: liver failure and received treatment. White blocks represent susceptible, including those spontaneously cleared and cured from HCV. (B) HCV care cascade and HCV testing pathways. Orange arrows indicate the movement influenced by the testing scenarios. (C) Population groups and transitions. The greek symbols represent the parameters related to transition between compartments.
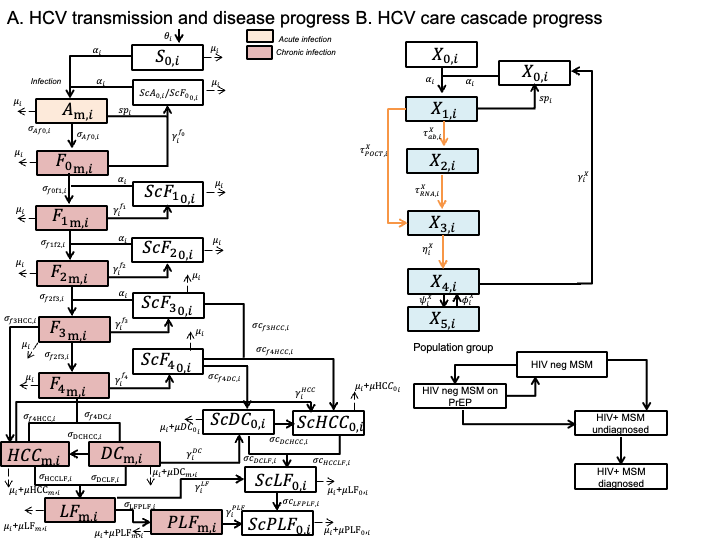


**[Figure S2.](#FigureS2) Calibration of model to the number of MSM on PrEP and MSM living with HIV.** Black data points (whiskers) represent notification data from Taiwan CDC notification database with an assumed range (represented as whiskers). The solid lines are the corresponding best estimates from the model simulations and the shading shows the 95% percentile interval for the 1000 simulations.


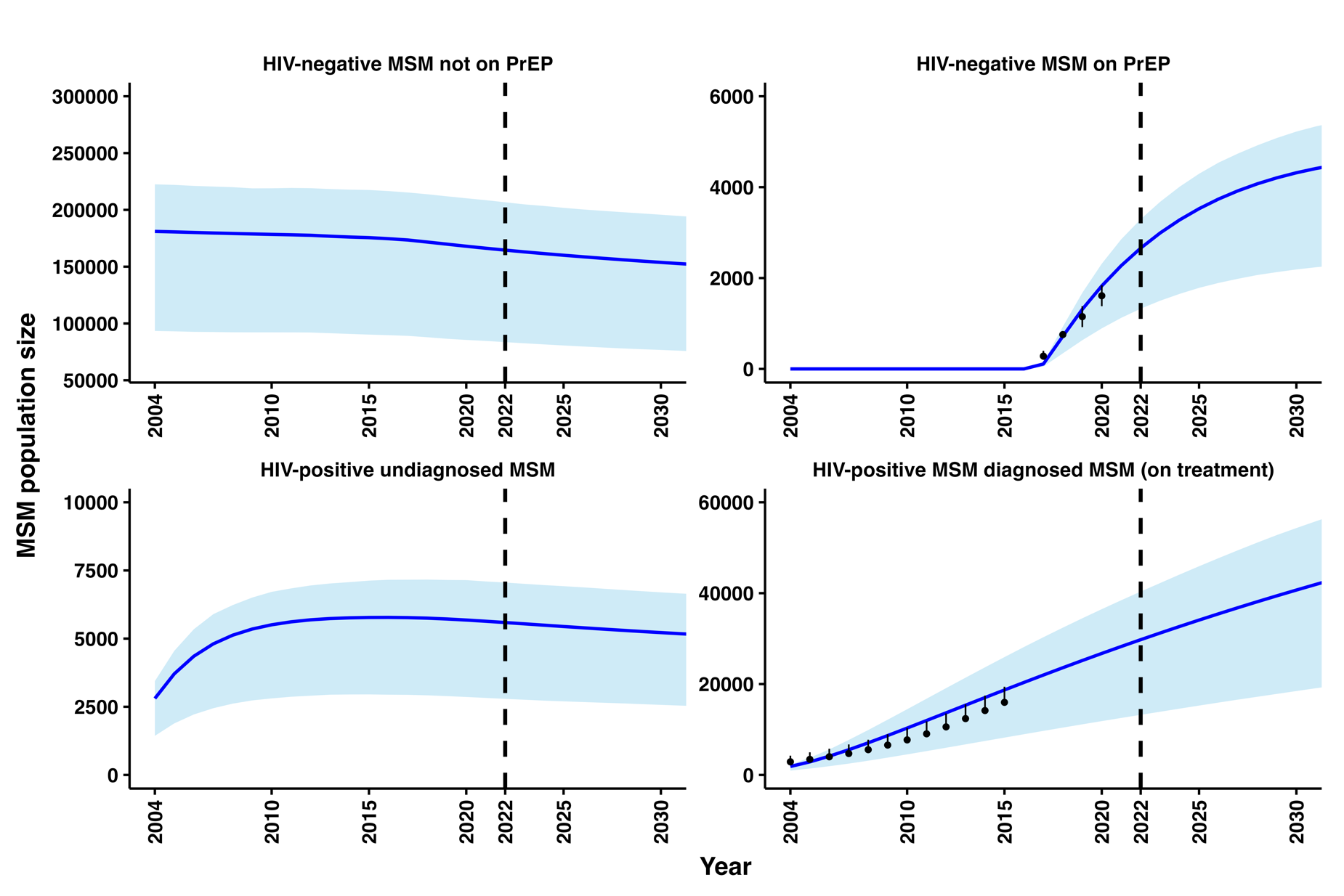


**[Figure S3.](#FigureS3) Calibration of model to the (A) HIV prevalence, (B) HIV incidence, (C) proportion of HIV diagnosed, and (D) number of annually HIV diagnosed.** Black data points (whiskers) represent the point estimates from the extracted studies with an 95% confidence interval (represented as whiskers) in (A), (B), and (C). Black data points (whiskers) represent notification data from Taiwan CDC notification database with an assumed range (represented as whiskers) in (D). The solid lines are the corresponding best estimates from the model simulations and the shading shows the 95% percentile interval for the 1000 simulations.


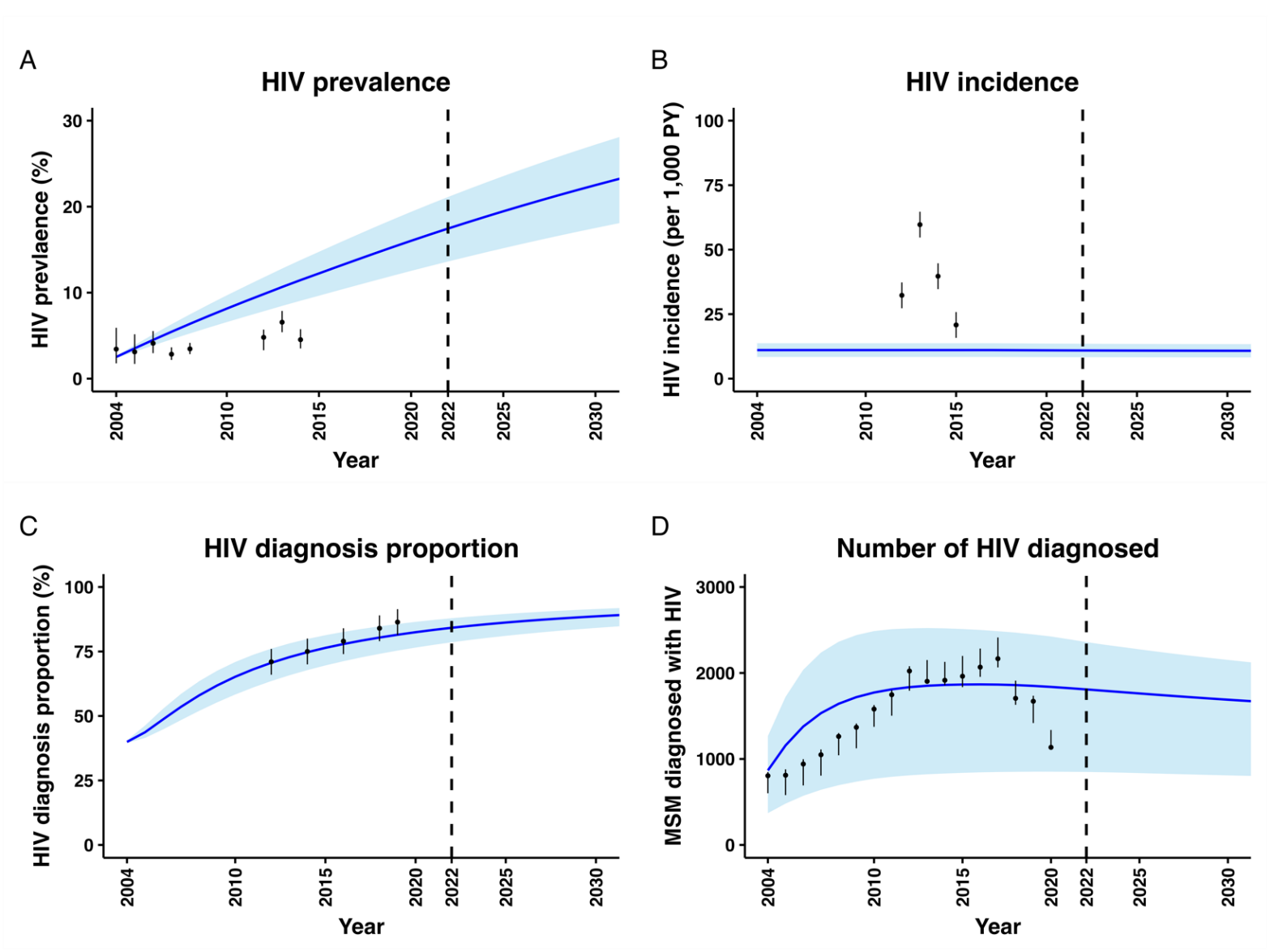


**[Figure S4.](#FigureS4) Simulated HCV prevalence (A) and HCV incidence (B) in the overall MSM population in Taiwan.** There are no specific data available to inform the model calibration. The solid lines are the corresponding best estimates from the model simulations and the shading shows the 95% percentile interval for the 1000 simulations.


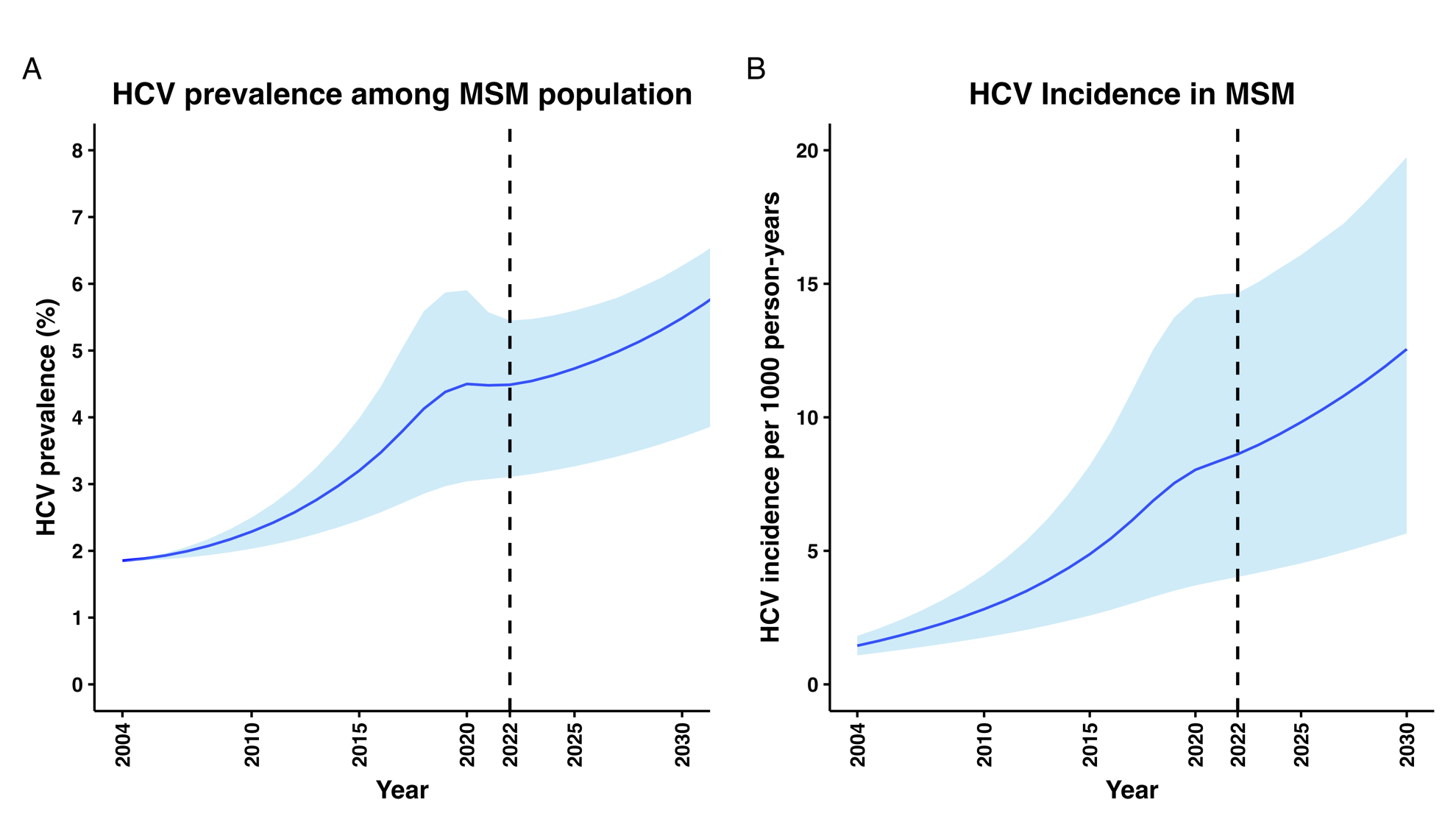


**[Figure S5.](#FigureS5) Calibration of model to (A) HCV prevalence and (B) in MSM subpopulations.** Black data points (whiskers) represent the point estimates from the extracted literature with an 95% confidence interval (represented as whiskers). The solid lines are the corresponding best estimates from the model simulations and the shading shows the 95% percentile interval for the 1000 simulations.


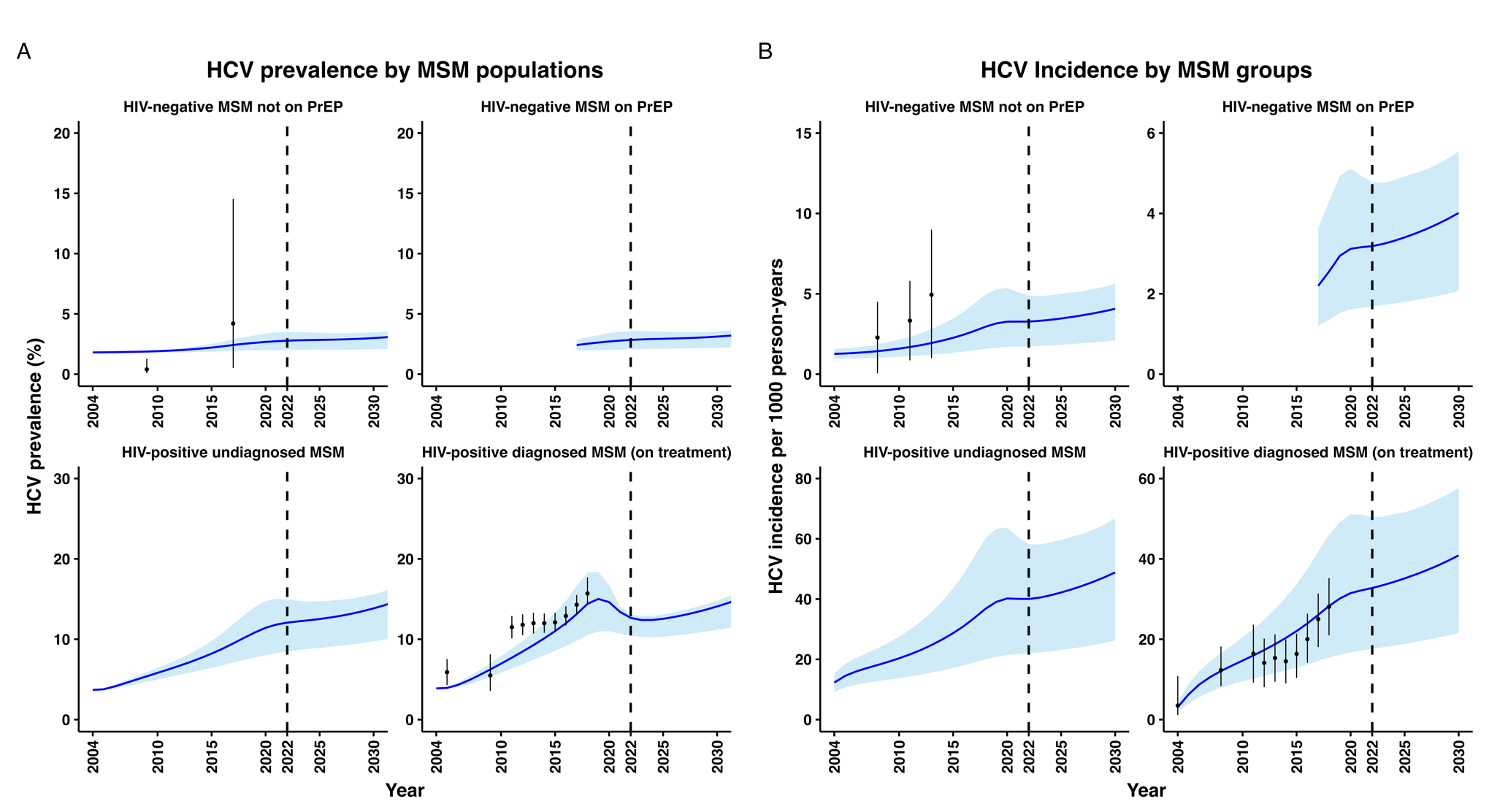


**[Figure S6.](#FigureS6) Calibration of model to (A) cumulative number of HCV treatment initiated and (B) cumulative number of HCV SVR in overall MSM.** Black data points (whiskers) represent mean estimates from data of Taiwan National Health insurance database with an 95% confidence interval (represented as whiskers). The solid lines are the corresponding best estimates from the model simulations and the shading shows the 95% percentile interval for the 1000 simulations.


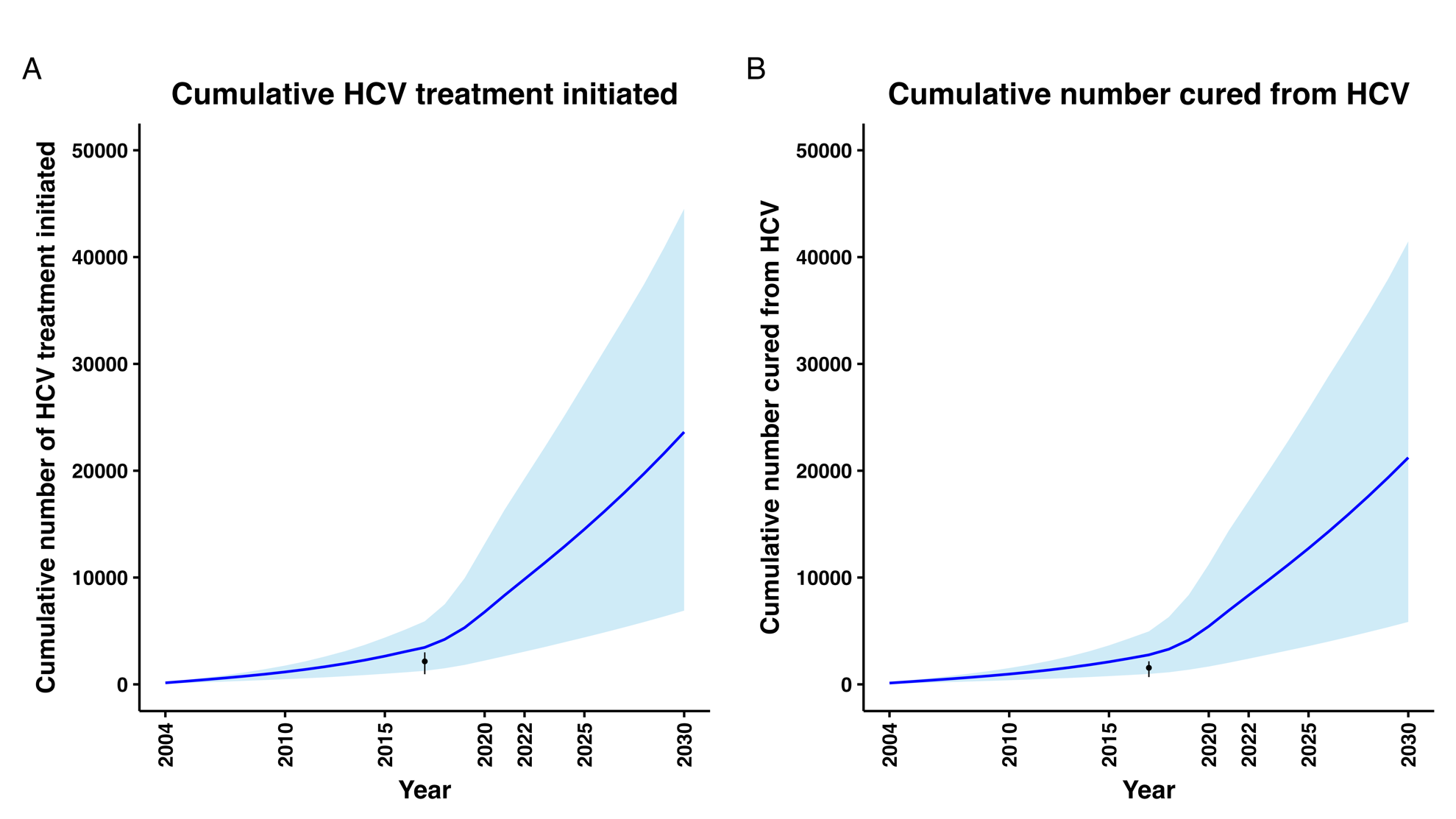


**[Figure S7.](#FigureS7) Simulated cumulative number of HCV treatment initiated (A) and cumulative number of HCV SVR (B) in MSM subpopulations in Taiwan.** There are no specific data available to inform the model calibration. The solid lines are the corresponding best estimates from the model simulations and the shading shows the 95% percentile interval for the 1000 simulations.


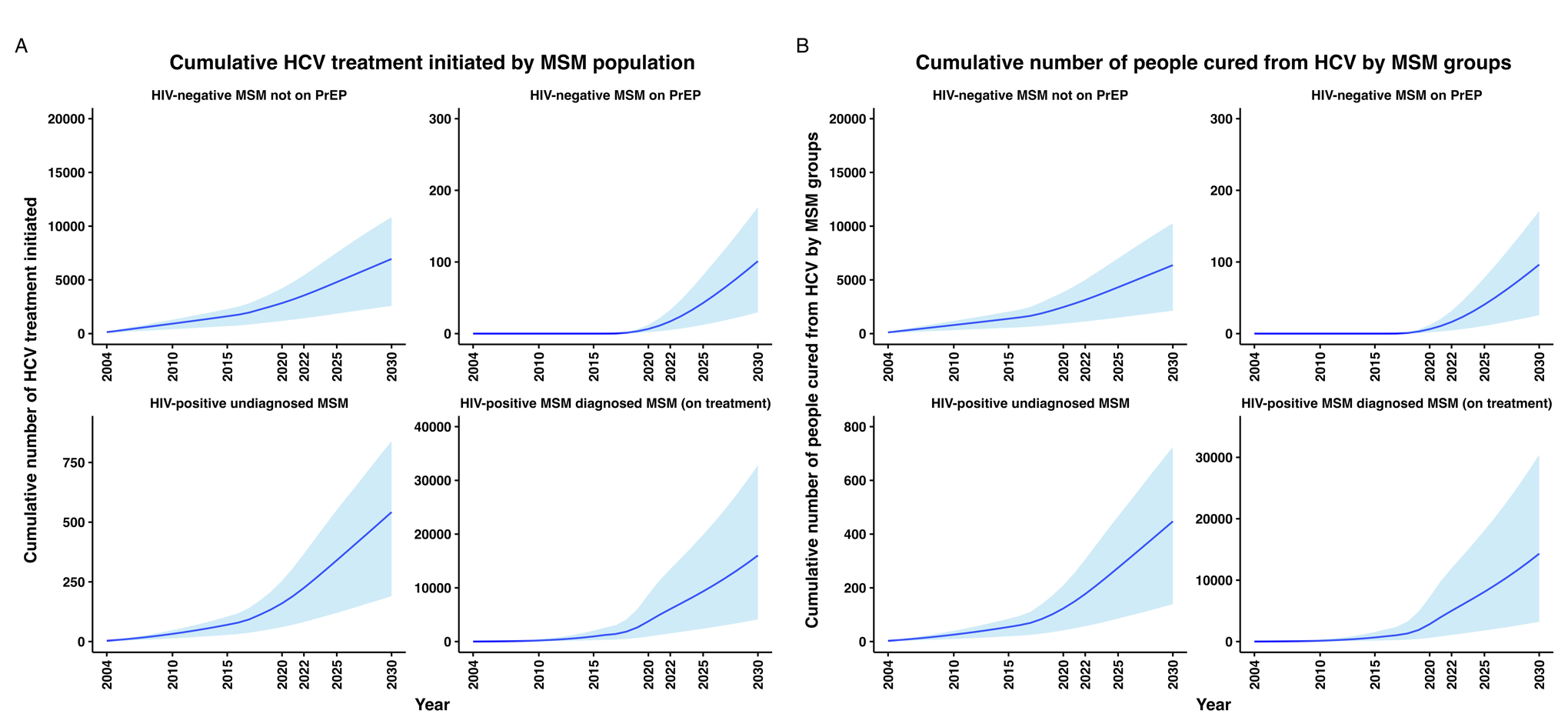


# **3. Additional results**

**Figure S8** to **Figure S12** show the impact of simplified HCV testing scenarios on HCV epidemic among MSM subpopulations.

**[Figure S8.](#FigureS8) Impact of simplified HCV testing strategies on HCV prevalence in MSM subpopulations compared to status quo 2015-2030 in Taiwan.** The lines correspond to median of the simulations.


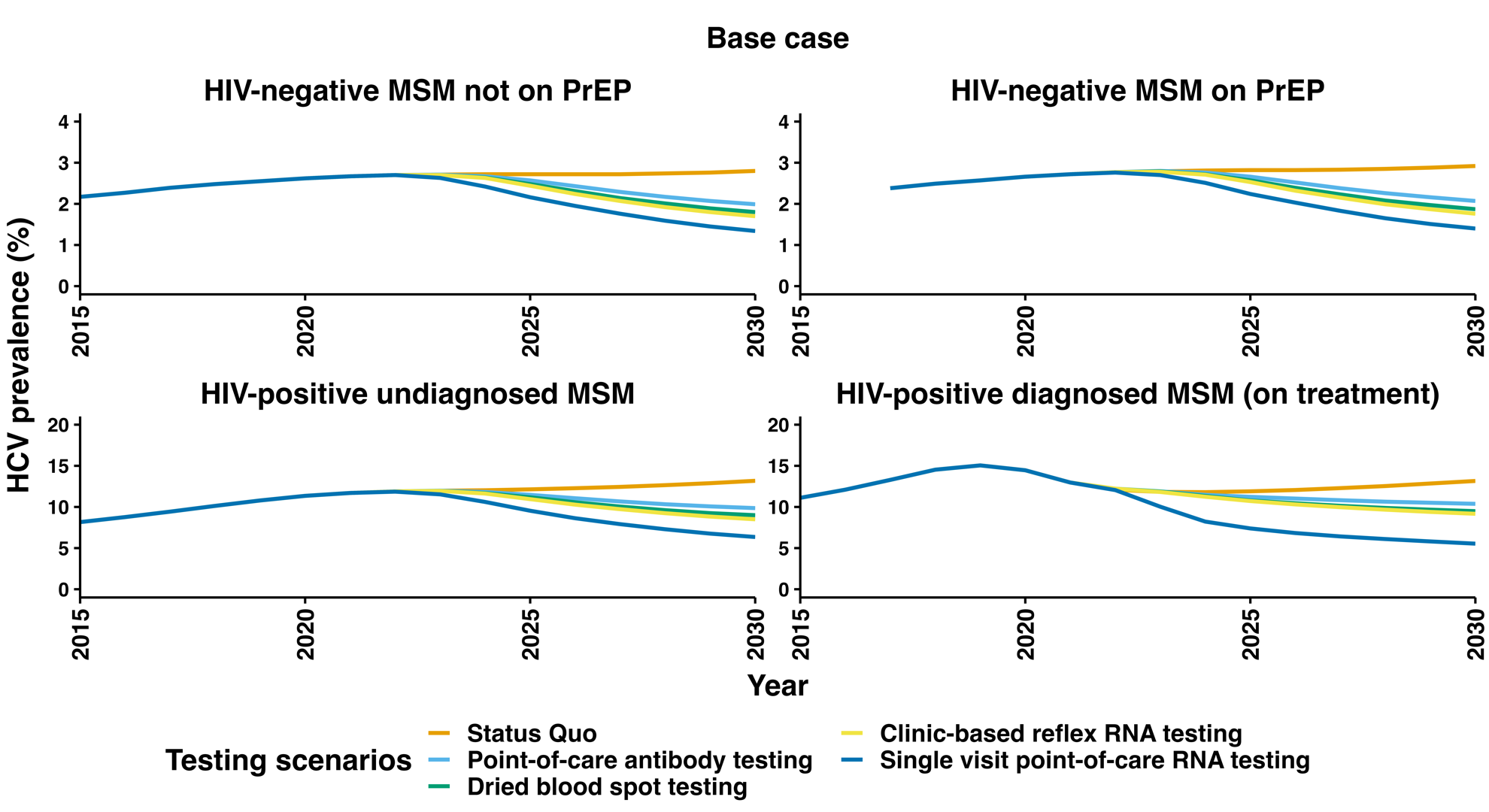


**[Figure S9.](#FigureS9) Impact of simplified HCV testing strategies on HCV incidence in overall MSM compared to status quo 2015-2030 in Taiwan.** The lines correspond to median of the simulations. The dashed line represents the WHO elimination threshold.


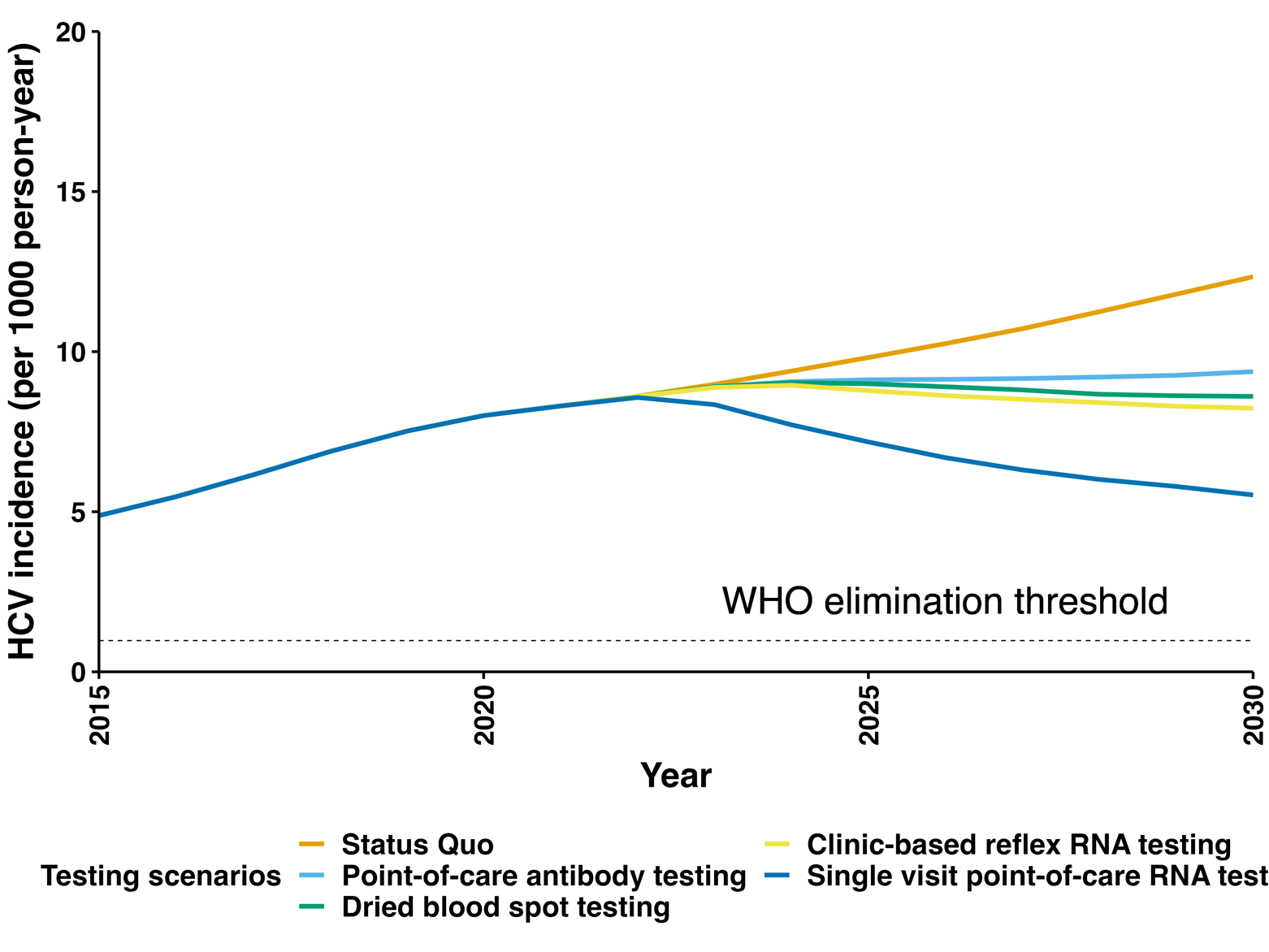


**[Figure S10.](#FigureS10) Impact of simplified HCV testing strategies on HCV incidence in MSM subpopulations compared to status quo 2015-2030 in Taiwan.** The lines correspond to median of the simulations. The dashed line represents WHO elimination threshold.
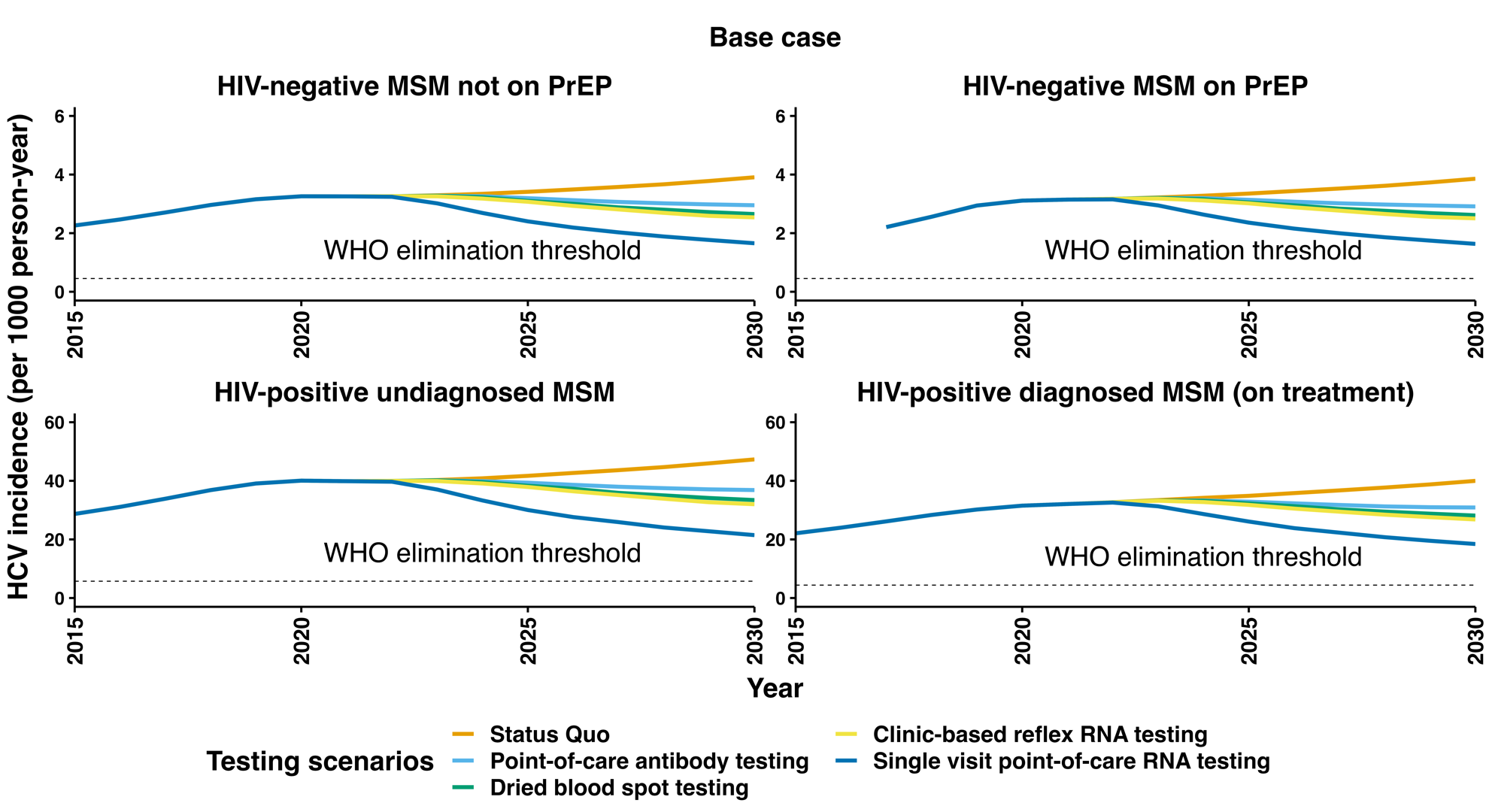


**[Figure S11.](#FigureS11)** **Impact of simplified HCV testing strategies on number of MSM living with chronic HCV in MSM subpopulations compared to status quo 2015-2030 in Taiwan.** The lines correspond to median of the simulations.


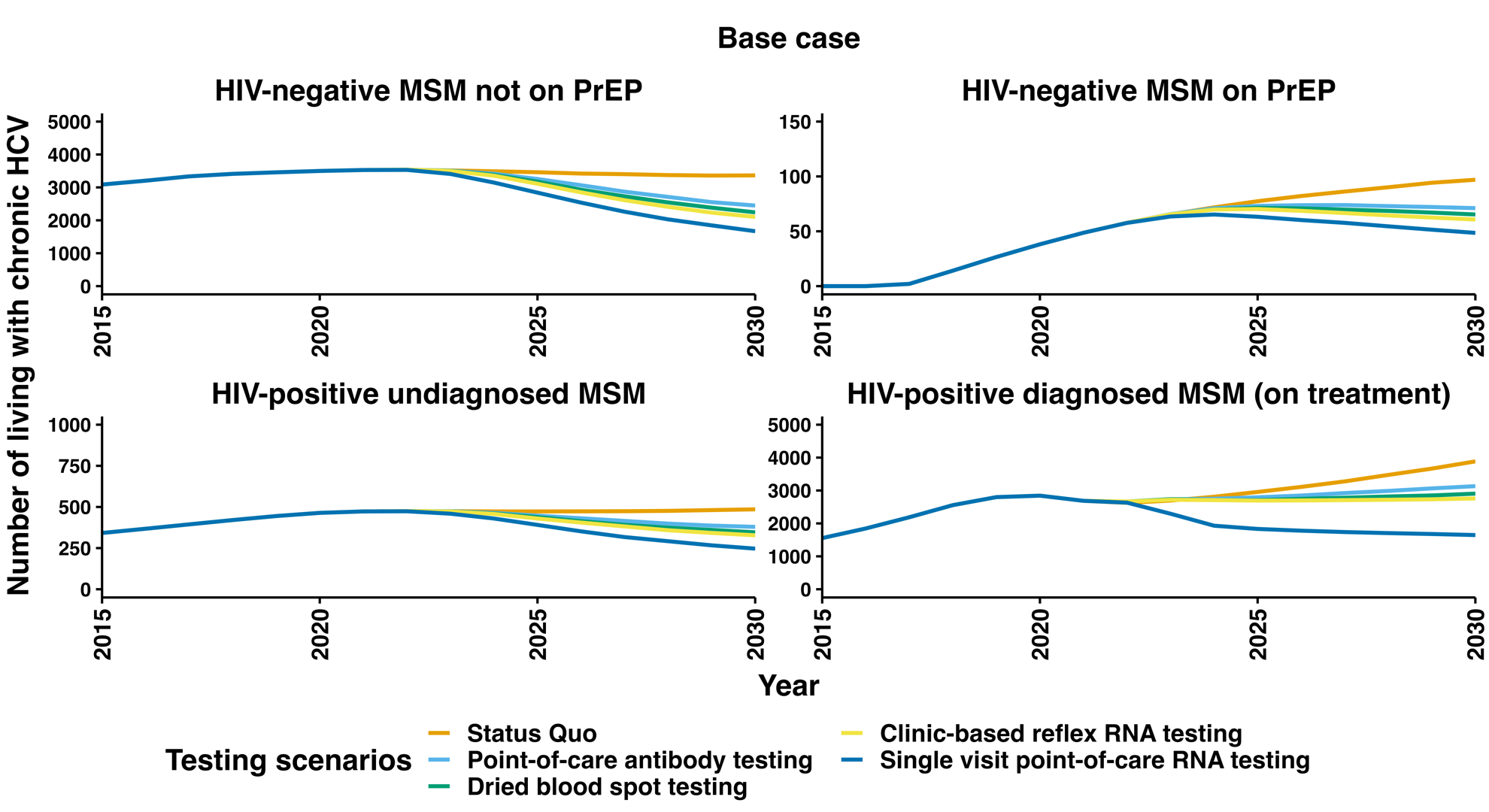


**[Figure S12.](#FigureS12) Impact of simplified HCV testing strategies on annual incident cases of HCV infection in MSM subpopulations compared to status quo 2015-2030 in Taiwan.** The lines correspond to median of the simulations.


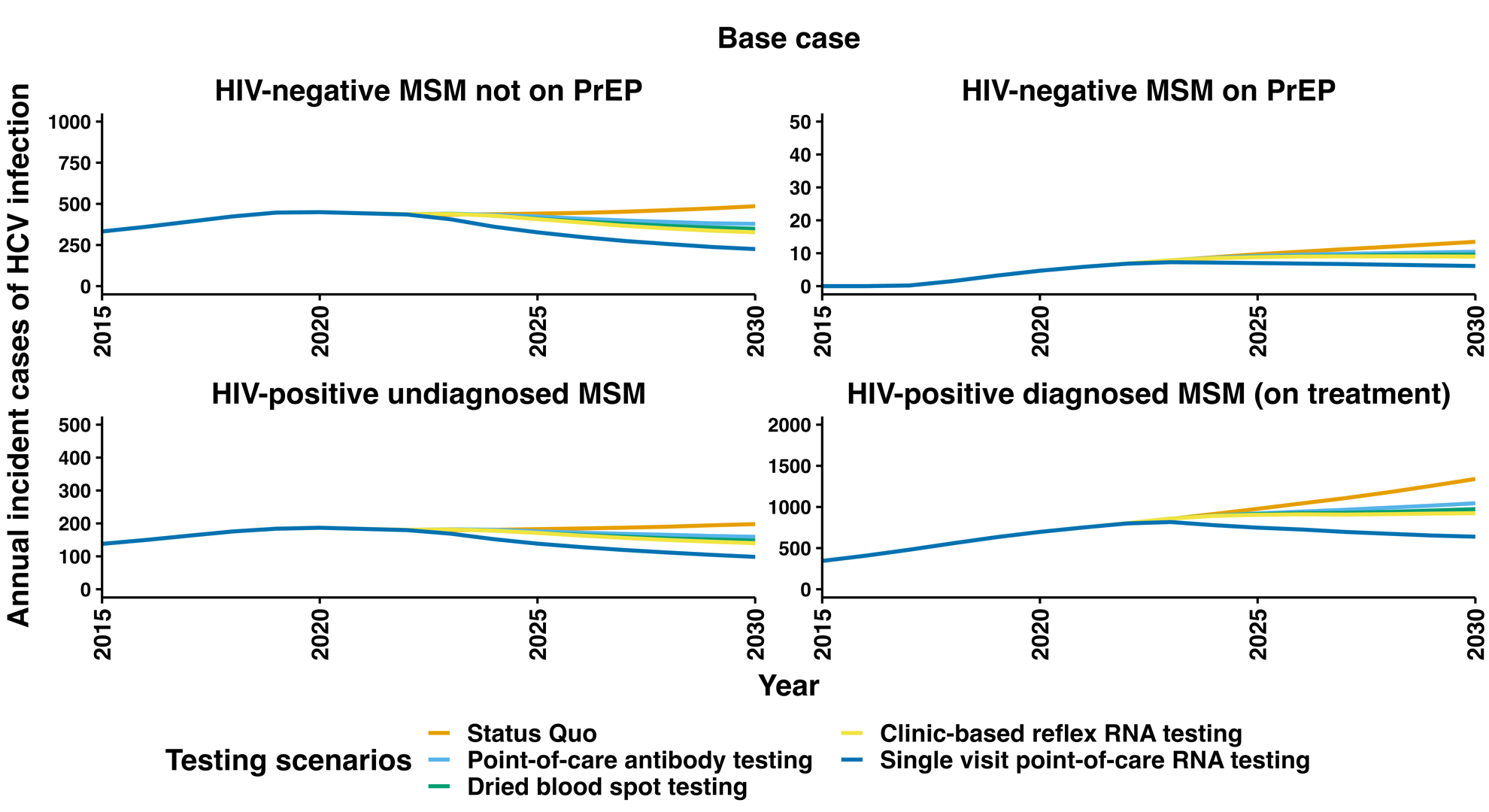


# **4. Sensitivity analysis**

We ran a series of scenarios varying associated model parameters for the simplified HCV testing scenarios to explore the impact of strategy scale-up time frame, effect of testing strategies, reinfection, and targeted subpopulations. We provided the details of sensitivity analysis and the results of this one-way analysis.

Model parameters for the simplified HCV testing scenarios varied:

1. scale-up time frame:
   1. Base case scenario: 2 years (2022-2024)
   2. Sensitivity analysis scenarios
      1. Optimized scale-up: scale-up HCV testing strategies from 2022-2023 (1-year).
      2. Pessimistic scale-up: scale-up HCV testing strategies from 2022-2027 (5-year).
2. the effect of HCV testing strategies:
   1. Base case scenario:
3. point-of-care testing antibody testing
   - - 1. 98% of MSM diagnosed with HIV and on HIV treatment received HCV antibody testing.
       2. 74.8% of each rest MSM subpopulations received HCV antibody testing.
4. dried blood spot testing
   - - 1. 98% of MSM diagnosed with HIV and on HIV treatment received HCV antibody testing.
       2. 52.3% of each rest MSM subpopulations received HCV antibody testing.
5. clinic-based RNA testing
   - - 1. 98% of MSM diagnosed with HIV and on HIV treatment received HCV antibody testing.
       2. 75.3% of each rest MSM subpopulations received HCV antibody testing.
6. point-of-care RNA testing*
   - - 1. 98% of MSM diagnosed with HIV and on HIV treatment received HCV RNA testing.
       2. 36.8% of each rest MSM subpopulations received HCV RNA testing.

* Point-of-care RNA testing strategy varied parameter of HCV RNA testing since it did not require HCV antibody testing.

- 1. Sensitivity analysis scenarios:

We test two different scaled-up time frames of HCV testing strategies to compare the outcome of the base case scenario. HCV testing for MSM diagnosed with HIV and on HIV treatment remained as same as in the base case scenario.

1. Optimized impact of HCV testing

We increased either HCV antibody testing or HCV RNA testing (if HCV antibody testing was not required in the testing scenario) to 80% for the rest of MSM subpopulations across four testing strategies.

1. Pessimistic impact of HCV testing

We increased either HCV antibody testing or HCV RNA testing (if HCV antibody testing was not required in the testing scenario) to 80% for the rest of MSM subpopulations across four testing strategies.

1. reinfection rate
   1. Base case scenario: same as primary infection rate.
   2. Sensitivity analysis scenarios:
      1. Reinfection rate equals to 1·5 fold of primary infection rate from year of 2022.
      2. no reinfection occurred since the year 2022.
2. targeted population
   1. Base case scenario: entire MSM population.
   2. Sensitivity analysis scenarios:
      1. prioritizing HCV testing strategies to MSM subgroups who are regularly engaged with HIV prevention and care services.
3. **Effect of changing scale-up timeframe**

**[Figure S13.](#FigureS13) Effect of changing scale-up timeframe on HCV prevalence in Simplified HCV testing strategies among MSM subgroups compared to status quo 2015-2030 in Taiwan.** Upper: scaling up simplified HCV testing strategies over a 5-year period, 2022-2027; Lower: scaling up simplified HCV testing strategies over a 1-year period, 2022-2023).


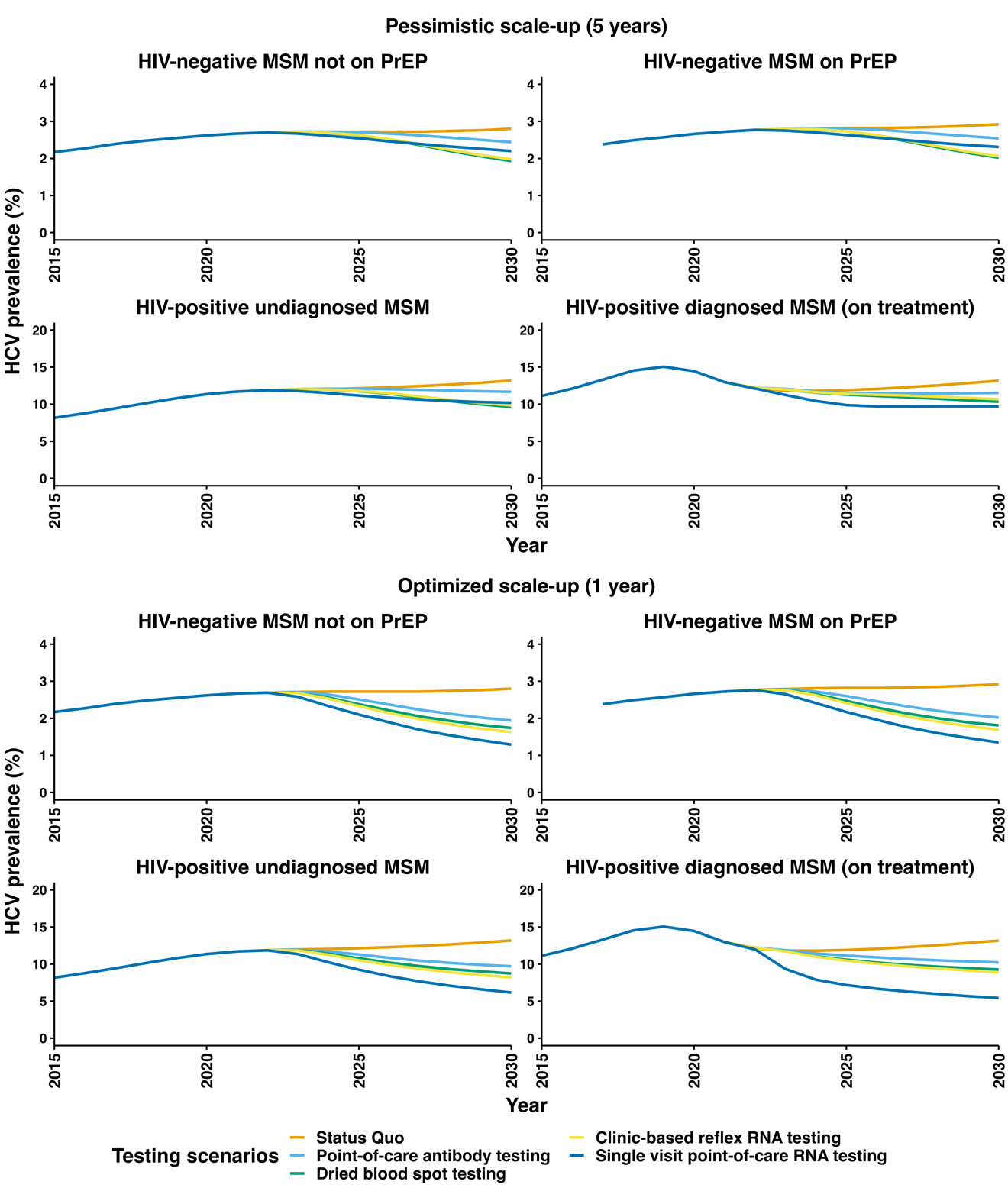


**[Figure S14.](#FigureS14) Effect of changing scale-up timeframe on HCV incidence in Simplified HCV testing strategies among MSM subgroups compared to status quo 2015-2030 in Taiwan.** Upper: scaling up simplified HCV testing strategies over a 5-year period, 2022-2027; Lower: scaling up simplified HCV testing strategies over a 1-year period, 2022-2023).

**
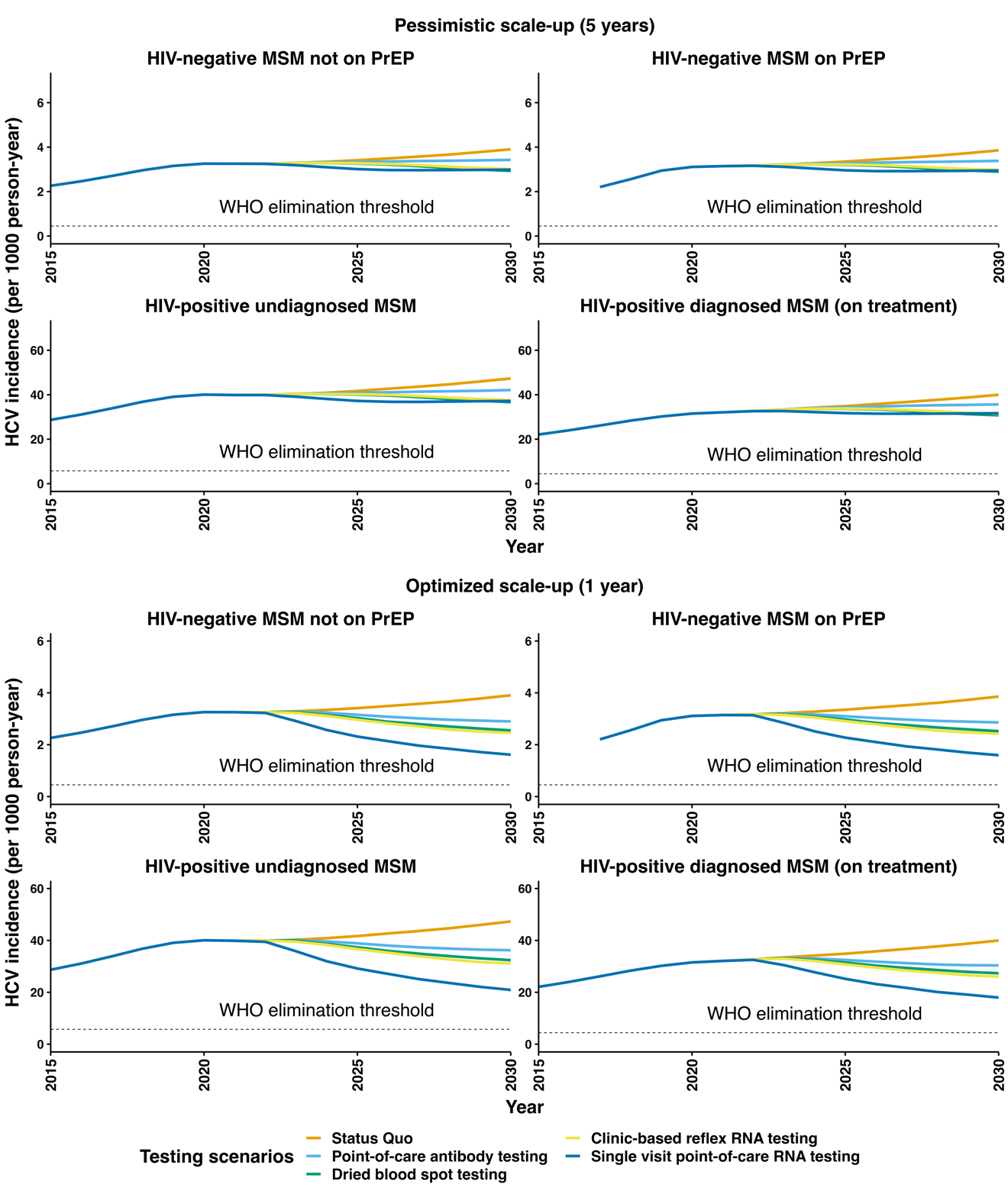
**

1. **Effect of varying effect of simplified HCV testing strategies**

**[Figure S15.](#FigureS15) Effect of varying effect of simplified HCV testing strategies on HCV prevalence in Simplified HCV testing strategies among MSM subgroups compared to status quo 2015-2030 in Taiwan.** Upper: pessimistic impact on HCV diagnosis (30%); Lower: optimistic impact on HCV diagnosis (80%).


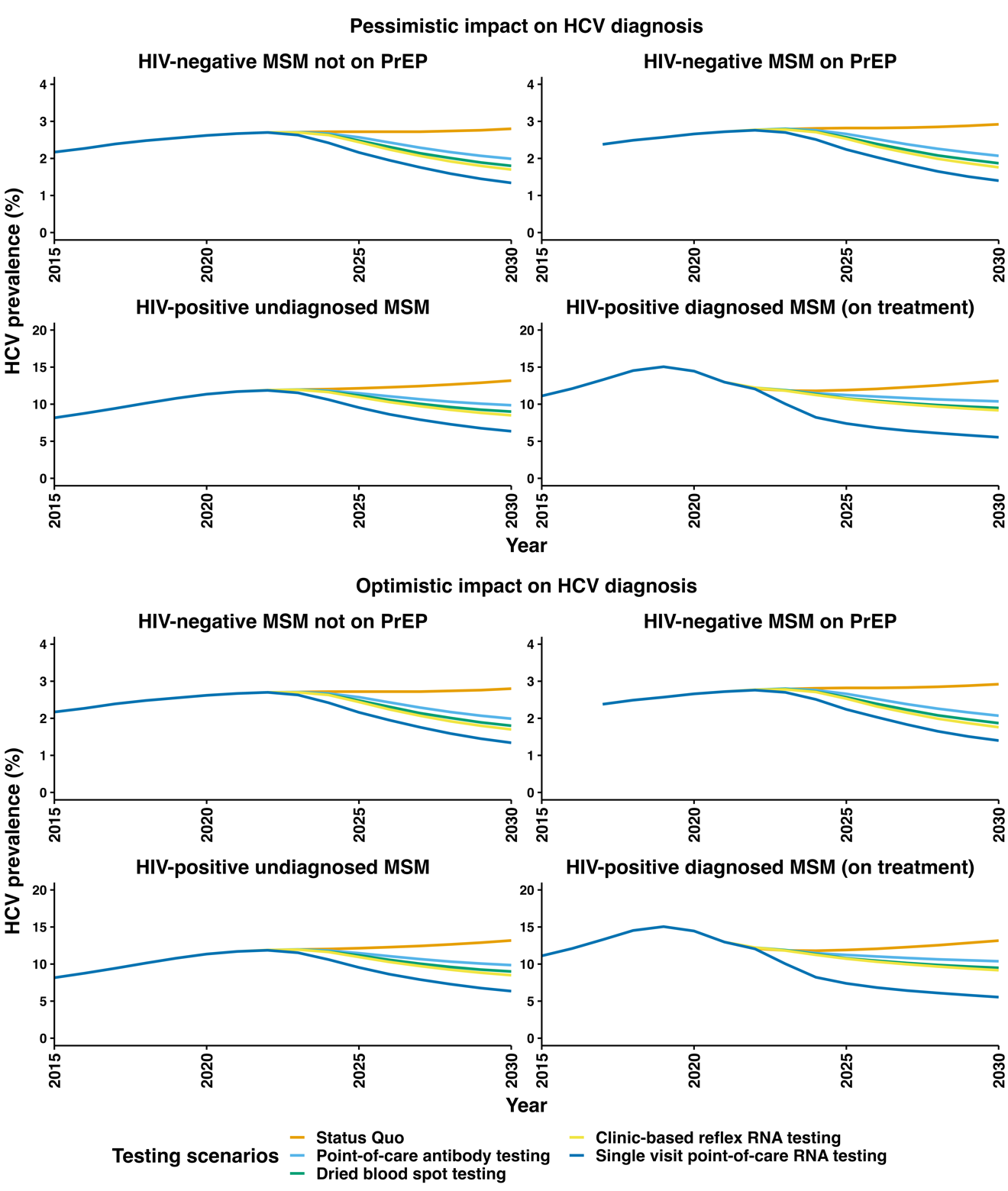


**[Figure S16.](#FigureS16) Effect of varying effect of POINT-OF-CARE testing on HCV incidence in Simplified HCV testing strategies among MSM subgroups compared to status quo 2015-2030 in Taiwan.** Upper: pessimistic impact on HCV diagnosis (30%); Lower: optimistic impact on HCV diagnosis (80%).

**
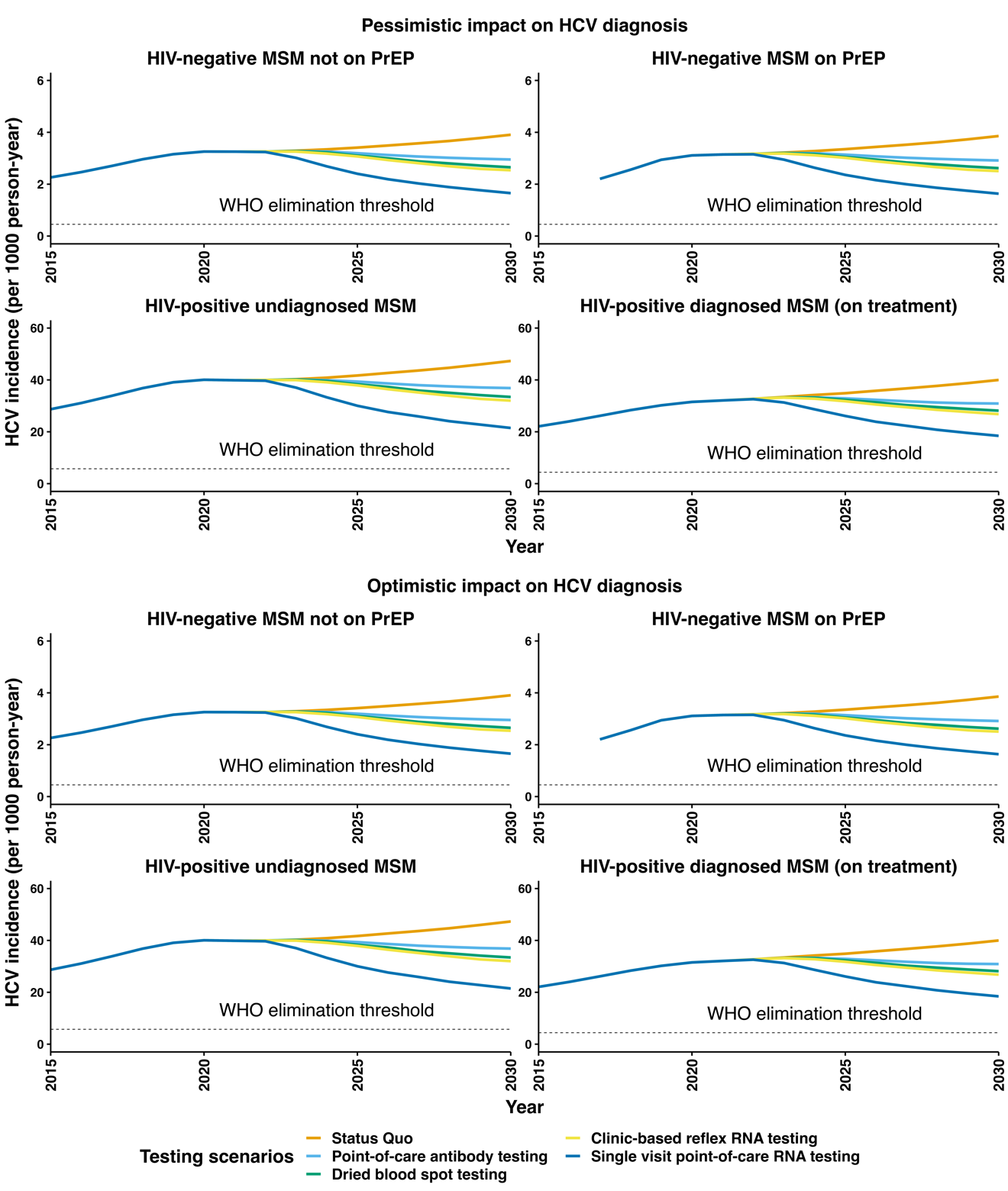
**

1. **Effect of changing reinfection rate**

**[Figure S17.](#FigureS17) Effect of changing reinfection rate on HCV prevalence in Simplified HCV testing strategies among MSM subgroups compared to status quo 2015-2030 in Taiwan.** Upper: pessimistic impact on HCV diagnosis (30%); Lower: optimistic impact on HCV diagnosis (80%).


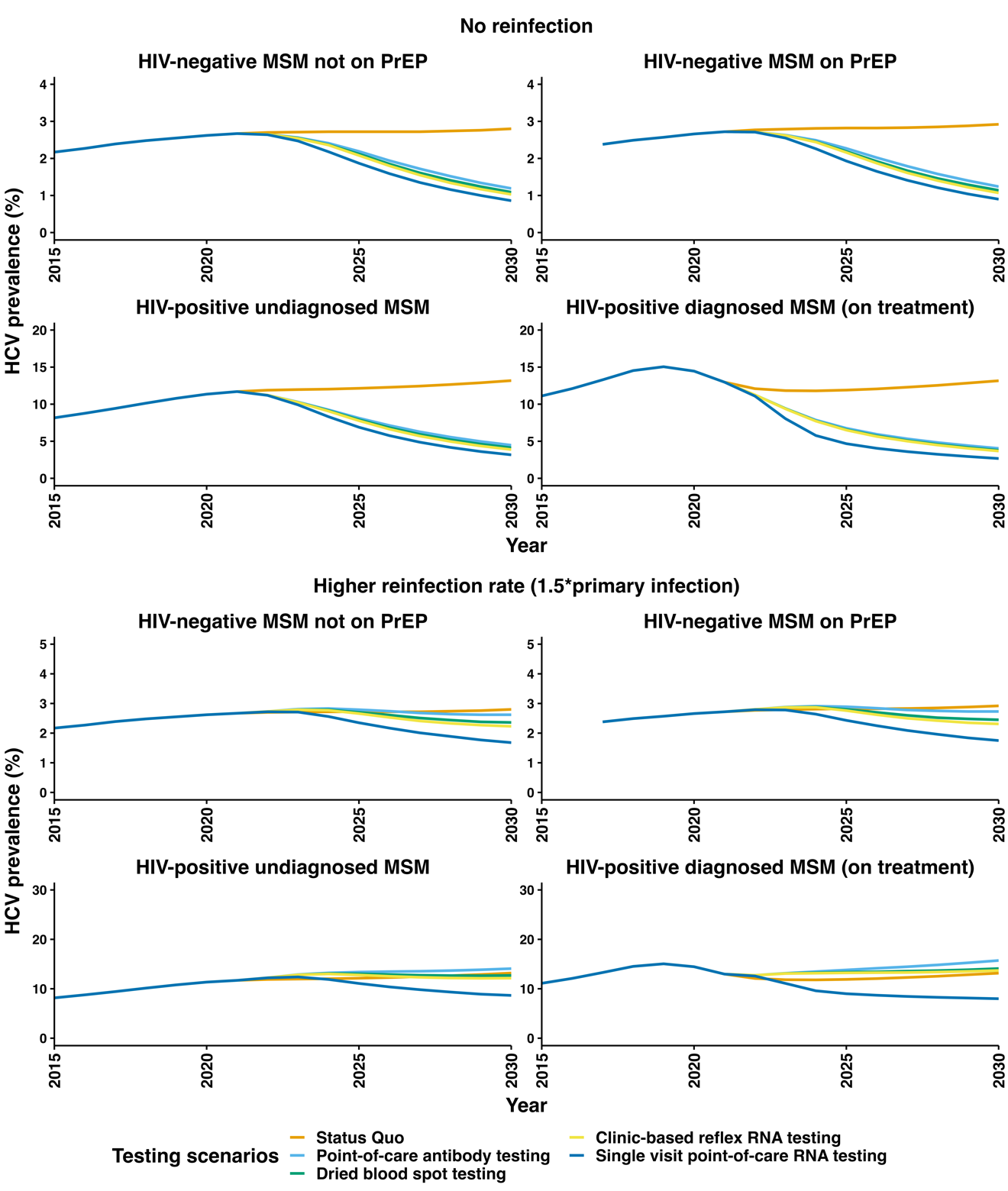


**[Figure S18.](#FigureS18) Effect of changing reinfection rate on HCV incidence in Simplified HCV testing strategies among MSM subgroups compared to status quo 2015-2030 in Taiwan.** Upper: pessimistic impact on HCV diagnosis (30%); Lower: optimistic impact on HCV diagnosis (80%).


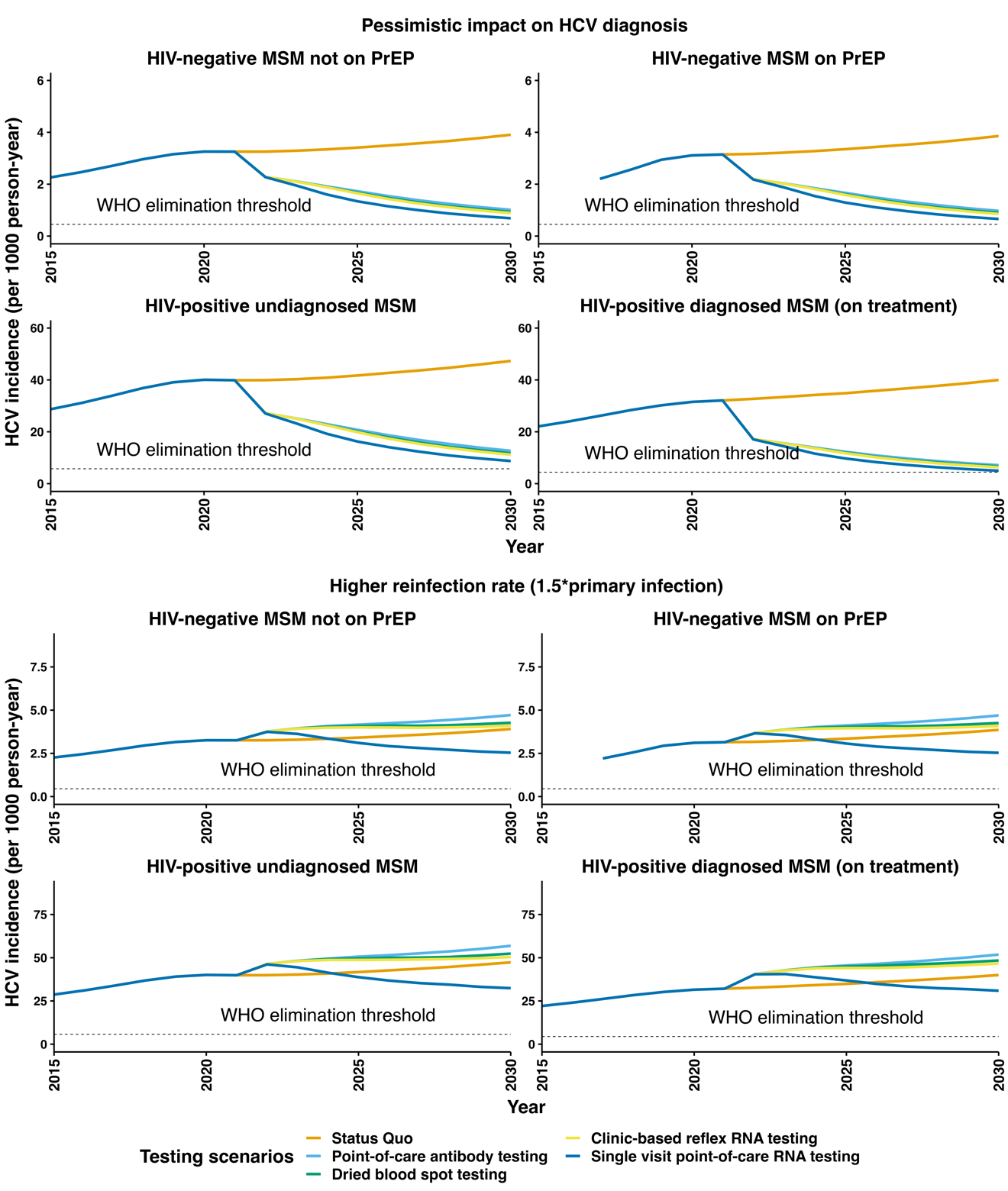


1. **Effect of prioritized MSM who regularly engaged with HIV prevention and care services.**

**[Figure S19.](#FigureS19) Effect of prioritized HIV negative MSM on PrEP and HIV diagnosed MSM on HCV prevalence in simplified HCV testing strategies among MSM subgroups compared to status quo 2015-2030 in Taiwan.**

**
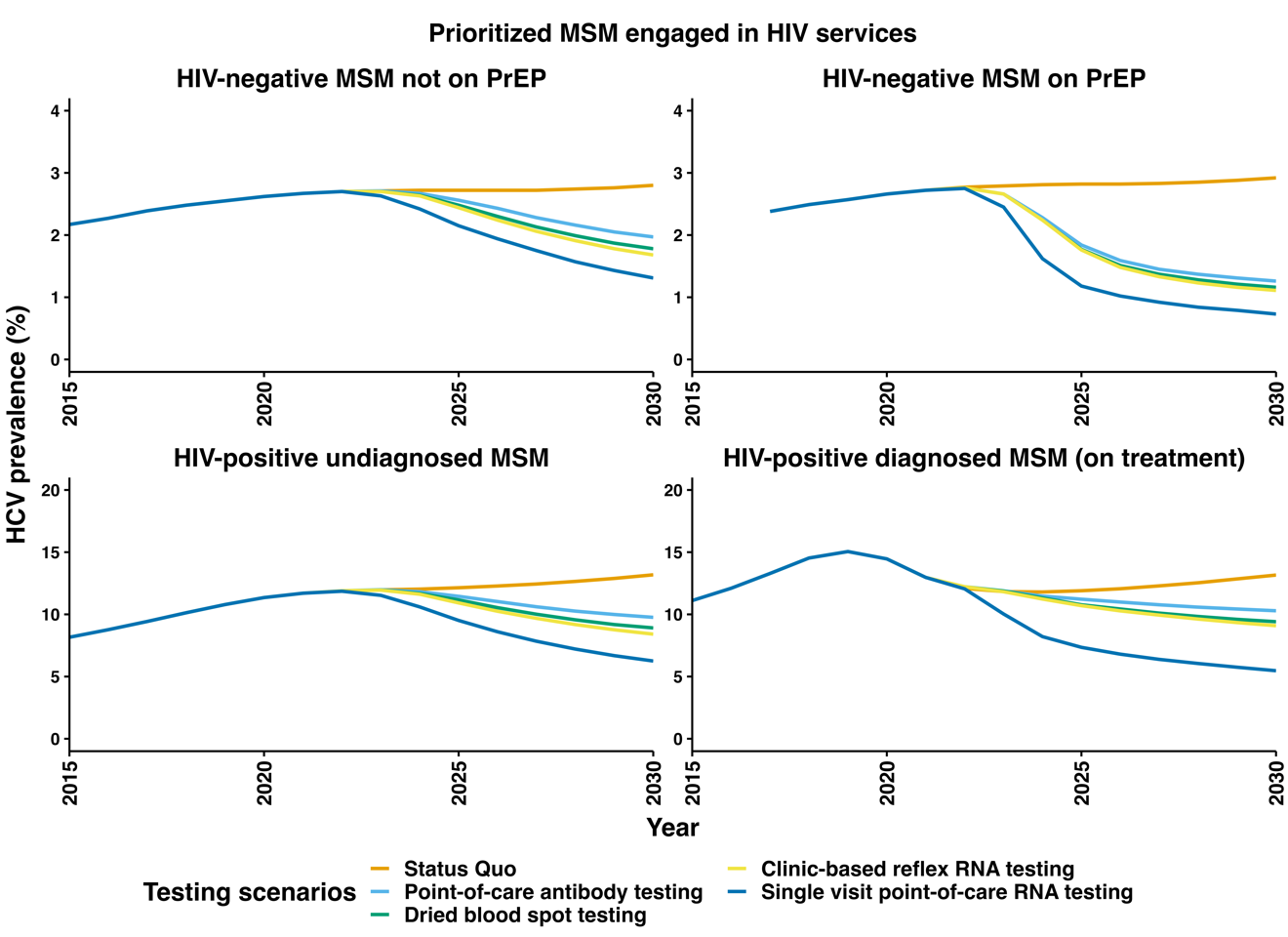
**

**[Figure S20.](#FigureS20) Effect of prioritized HIV negative MSM on PrEP and HIV diagnosed MSM on HCV prevalence in simplified HCV testing strategies among MSM subgroups compared to status quo 2015-2030 in Taiwan.**
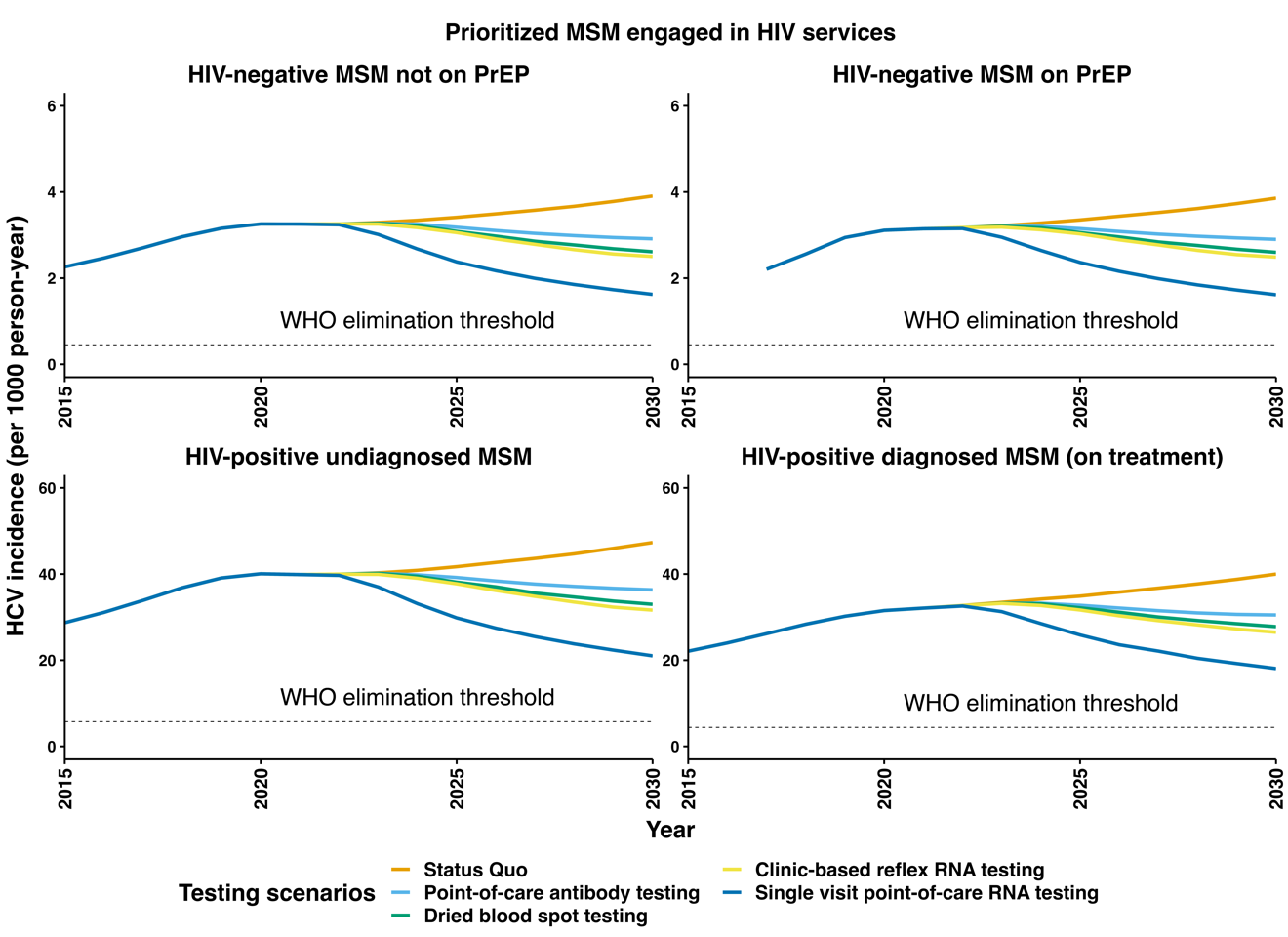


**[Figure S21.](#FigureS21) Cumulative new HCV cases over 2022-2030 for simplified HCV testing strategies among MSM subgroups in Taiwan for each sensitivity analysis scenario (best estimates with 95% percentile interval from 1000 simulations).**


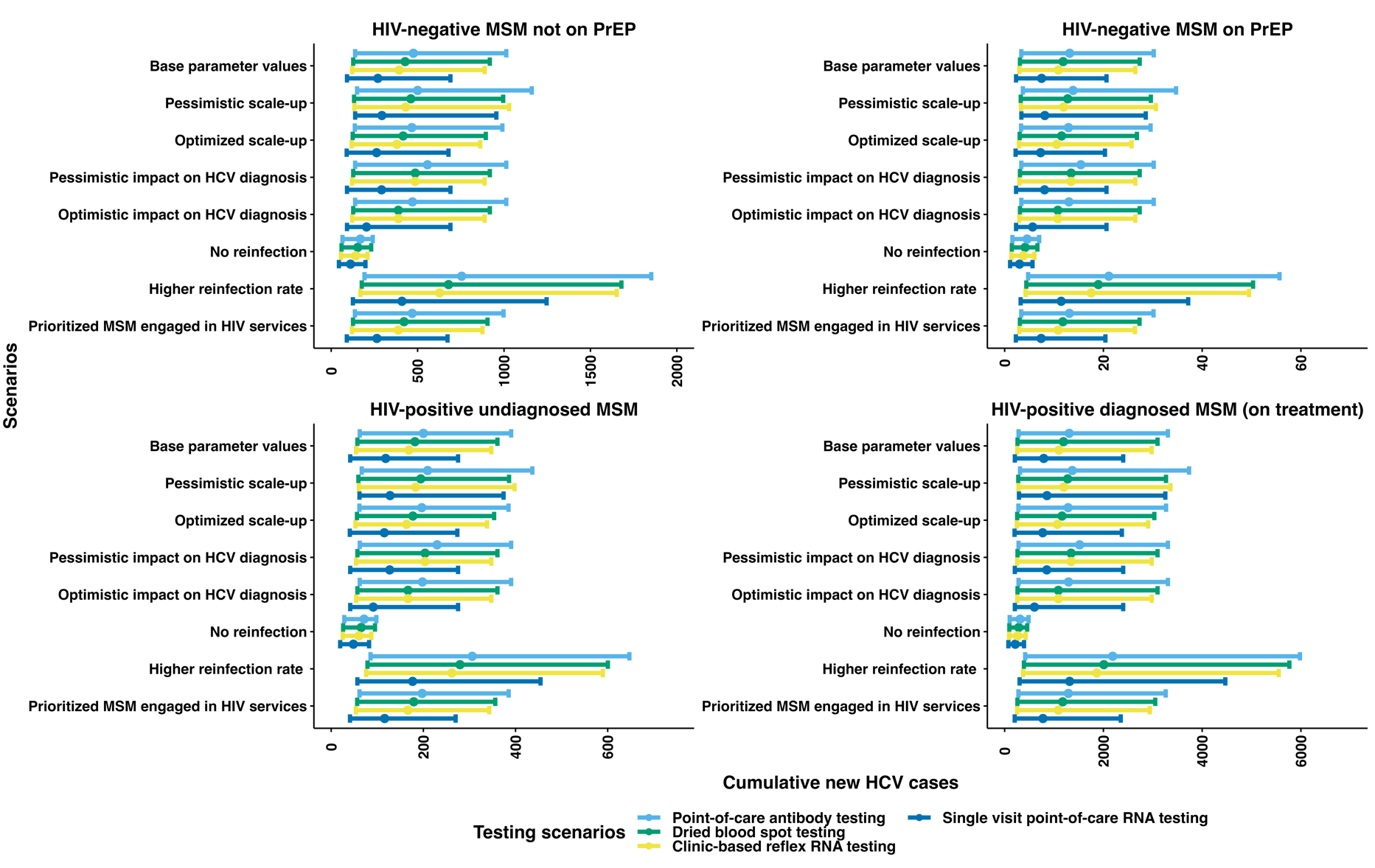


# **5. References for supplementary material**

1 Wu S-B, Huang Y-C, Huang Y-F, Huang J-C. Estimating HIV incidence, prevalence, and percent of undiagnosed infections in Taiwan using CD4 data. *Journal of the Formosan Medical Association* 2022; **121**: 482–9.

2 Smit C, Boyd A, Rijnders BJA, *et al.* HCV micro-elimination in individuals with HIV in the Netherlands 4 years after universal access to direct-acting antivirals: a retrospective cohort study. *The Lancet HIV* 2021; **8**: e96–105.

3 Wells JE, McGee MA, Beautrais AL. Multiple aspects of sexual orientation: Prevalence and sociodemographic correlates in a New Zealand national survey. *Archives of Sexual Behavior* 2011; **40**: 155–68.

4 Sexual identity, UK - Office for National Statistics. https://www.ons.gov.uk/peoplepopulationandcommunity/culturalidentity/sexuality/bulletins/sexualidentityuk/2016 (accessed Aug 20, 2022).

5 Rich AJ, Lachowsky NJ, Sereda P, *et al.* Estimating the size of the MSM population in Metro Vancouver, Canada, using multiple methods and diverse data sources. *Journal of Urban Health* 2018; **95**: 188–95.

6 Li W-Y, Chen M, Huang S-W, *et al.* Molecular epidemiology of HIV-1 infection among men who have sex with men in Taiwan from 2013 to 2015. *PLOS ONE* 2018; **13**: e0202622–e0202622.

7 Tseng F-C, Ko N-Y, Lee H-C, Wu C-J, Hung C-C, Ko W-C. HIV risk profiles differed by gender and experience of men who had sex with men among attendees of anonymous voluntary counseling and testing in Taiwan. *http://dx.doi.org/101080/095401212012749338* 2013; **25**: 1092–101.

8 Huang S-W, Wang S-F, Cowo AE, *et al.* Molecular epidemiology of HIV-1 infection among men who have sex with men in Taiwan in 2012. *PLoS One* 2015; **10**: e0128266.

9 Taiwan CDC. Updated HIV/AIDS statistics in Taiwan. Centers for Disease Control. .

10 Wu H, Strong C, Ku S, *et al.* Syphilis acquisition and dosing schedule for pre-exposure prophylaxis (PrEP) users in Taiwan PrEP demonstration project. .

11 Wu H, Wen‐Wei Ku S, Chang HH, Li C, Ko N, Strong C. Imperfect adherence in real life: a prevention‐effective perspective on adherence to daily and event‐driven HIV pre‐exposure prophylaxis among men who have sex with men–a prospective cohort study in Taiwan. *Journal of the International AIDS Society* 2021; **24**: e25733.

12 Taiwan Hepatitis C Policy Guidelines 2018-2025. Ministry of Health and Welfare, Executive Yuan ROC (Taiwan), 2019.

13 Yu M-L, Dai C-Y, Lin Z-Y, *et al.* A randomized trial of 24- vs. 48-week courses of PEG interferon α-2b plus ribavirin for genotype-1b-infected chronic hepatitis C patients: a pilot study in Taiwan. *Liver International* 2006; **26**: 73–81.

14 Population statistics, National Statistic portal of Taiwan. https://www.stat.gov.tw/ct.asp?xItem=15409&CtNode=3622&mp=4 (accessed Aug 20, 2022).

15 Hsieh S-H, Perri PF. Estimating the proportion of non-heterosexuals in Taiwan using Christofides’ randomized response model: A comparison of different estimation methods. *Social Science Research* 2021; **93**: 102475.

16 Lo Tung. Impact of Early HIV Diagnosis on Quality-adjusted Life Expectancy in HIV-infected Men Who Having Sex with Men (MSM). 2015.

17 Smith DJ, Jordan AE, Frank M, Hagan H. Spontaneous viral clearance of hepatitis C virus (HCV) infection among people who inject drugs (PWID) and HIV-positive men who have sex with men (HIV+ MSM): a systematic review and meta-analysis. *BMC infectious diseases* 2016; **16**: 1–13.

18 Micallef J, Kaldor JM, Dore GJ. Spontaneous viral clearance following acute hepatitis C infection: a systematic review of longitudinal studies. *Journal of viral hepatitis* 2006; **13**: 34–41.

19 Mondelli MU, Cerino A, Cividini A. Acute hepatitis C: diagnosis and management. *Journal of Hepatology* 2005; **42**: S108–14.

20 Thein H-H, Yi Q, Dore GJ, Krahn MD. Natural history of hepatitis C virus infection in HIV-infected individuals and the impact of HIV in the era of highly active antiretroviral therapy: a meta-analysis. *Aids* 2008; **22**: 1979–91.

21 Thein H-H, Yi Q, Dore GJ, Krahn MD. Estimation of stage‐specific fibrosis progression rates in chronic hepatitis C virus infection: a meta‐analysis and meta‐regression. *Hepatology* 2008; **48**: 418–31.

22 Xu F, Moorman AC, Tong X, *et al.* All-cause mortality and progression risks to hepatic decompensation and hepatocellular carcinoma in patients infected with hepatitis C virus. *Clinical Infectious Diseases* 2016; **62**: 289–97.

23 López-Diéguez M, Montes ML, Pascual-Pareja JF, *et al.* The natural history of liver cirrhosis in HIV–hepatitis C virus-coinfected patients. *Aids* 2011; **25**: 899–904.

24 Townsend R, McEwan P, Kim R, Yuan Y. Structural Frameworks and Key Model Parameters in Cost-Effectiveness Analyses for Current and Future Treatments of Chronic Hepatitis C. *Value in Health* 2011; **14**: 1068–77.

25 Research NC in HE and C. Epidemiological and economical impact of potential increased hepatitis C treatment uptake in Australia. The University of New South Wales Sydney, 2010.

26 Thein H-H, Yi Q, Heathcote EJ, Krahn MD. Prognosis of hepatitis C virus‐infected Canadian post‐transfusion compensation claimant cohort. *Journal of viral hepatitis* 2009; **16**: 802–13.

27 National Centre in HIV Epidemiology and Clinical Research. HIV/AIDS, viral hepatitis and sexually transmissible infections in Australia Annual Surveillance Report 2009. National Centre in HIV Epidemiology and Clinical Research: The University of New South Wales, Sydney, NSW.

28 Pineda JA, Romero‐Gómez M, Díaz‐García F, *et al.* HIV coinfection shortens the survival of patients with hepatitis C virus‐related decompensated cirrhosis. *Hepatology* 2005; **41**: 779–89.

29 Baillargeon J, Snyder N, Soloway RD, *et al.* Hepatocellular Carcinoma Prevalence and Mortality in a Male State Prison Population. *Public Health Rep* 2009; **124**: 120–6.

30 Yeh M-L, Liang P-C, Tsai P-C, *et al.* Characteristics and Survival Outcomes of Hepatocellular Carcinoma Developed after HCV SVR. *Cancers* 2021; **13**: 3455.

31 Hernaez R, Kramer JR, Liu Y, *et al.* Prevalence and short-term mortality of acute-on-chronic liver failure: A national cohort study from the USA. *Journal of Hepatology* 2019; **70**: 639–47.

32 Merchante N, Rivero-Juárez A, Téllez F, *et al.* Sustained virological response to direct-acting antiviral regimens reduces the risk of hepatocellular carcinoma in HIV/HCV-coinfected patients with cirrhosis. *Journal of Antimicrobial Chemotherapy* 2018; **73**: 2435–43.

33 Nahon P, Bourcier V, Layese R, *et al.* Eradication of hepatitis C virus infection in patients with cirrhosis reduces risk of liver and non-liver complications. *Gastroenterology* 2017; **152**: 142-156. e2.

34 McDonald SA, Barclay ST, Innes HA, *et al.* Uptake of interferon‐free DAA therapy among HCV‐infected decompensated cirrhosis patients and evidence for decreased mortality. *Journal of Viral Hepatitis* 2021; **28**: 1246–55.

35 Dang H, Yeo YH, Yasuda S, *et al.* Cure with interferon‐free direct‐acting antiviral is associated with increased survival in patients with hepatitis C virus‐related hepatocellular carcinoma from both east and west. *Hepatology* 2020; **71**: 1910–22.

36 Lee C-Y, Wu P-H, Tsai J-J, Chen T-C, Chang K, Lu P-L. Cascade analysis of anonymous voluntary HIV counseling and testing among patients with HIV infection in Taiwan. *AIDS Patient Care and STDs* 2020; **34**: 303–15.

37 Taiwan AIDS Society. Guidelines for diagnosis and treatment of HIV/AIDS, 6th edition. 2020. http://www.aids-care.org.tw/journal/treatment.php.

38 Spradling PR, Tong X, Rupp LB, *et al.* Trends in HCV RNA Testing Among HCV Antibody–Positive Persons in Care, 2003–2010. *Clinical Infectious Diseases* 2014; **59**: 976–81.

39 Yu M-L, Yeh M-L, Tsai P-C, *et al.* Huge gap between clinical efficacy and community effectiveness in the treatment of chronic hepatitis C: a nationwide survey in Taiwan. *Medicine* 2015; **94**.

40 Liu C-H, Sheng W-H, Sun H-Y, *et al.* Peginterferon plus ribavirin for HIV-infected patients with treatment-naïve acute or chronic HCV infection in Taiwan: a prospective cohort study. *Scientific reports* 2015; **5**: 1–11.

41 Liu C-H, Yu M-L, Peng C-Y, *et al.* Real-world anti-viral treatment decisions among chronic hepatitis C patients in Taiwan: The INITIATE study. *Journal of the Formosan Medical Association* 2019; **118**: 1014–23.

42 Wu GH-M, Yang W-W, Liu C-L, *et al.* The epidemiological profile of chronic hepatitis C with advanced hepatic fibrosis regarding virus genotype in Taiwan: A nationwide study. *Journal of the Formosan Medical Association* 2021.

43 Macgregor L, Ward Z, Martin NK, *et al.* The cost‐effectiveness of case‐finding strategies for achieving hepatitis C elimination among men who have sex with men in the UK. *Journal of viral hepatitis* 2021; **28**: 897–908.

44 Wen P-H, Lu C-L, Strong C, *et al.* Demographic and urbanization disparities of liver transplantation in Taiwan. *International journal of environmental research and public health* 2018; **15**: 177.

45 Nitulescu R, Young J, Saeed S, *et al.* Variation in hepatitis C virus treatment uptake between Canadian centres in the era of direct-acting antivirals. *International Journal of Drug Policy* 2019; **65**: 41–9.

46 Salazar-Vizcaya L, Wandeler G, Fehr J, *et al.* Impact of Direct-Acting Antivirals on the Burden of HCV Infection Among Persons Who Inject Drugs and Men Who Have Sex With Men in the Swiss HIV Cohort Study. *Open Forum Infectious Diseases* 2018; **5**: ofy154.

47 Huang M-H, Sun H-Y, Ho S-Y, *et al.* Recently acquired hepatitis C virus infection among people living with human immunodeficiency virus at a university hospital in Taiwan. *World Journal of Gastroenterology* 2021; **27**: 6277.

48 Tsai J-C, Hung C-C, Chang S-Y, *et al.* Increasing incidence of recent hepatitis C virus infection among persons seeking voluntary counselling and testing for HIV and sexually transmitted infections in Taiwan. *BMJ open* 2015; **5**: e008406.

49 Su YC, Liu WC, Chang LH, *et al.* Incidence of recent HCV infection among persons seeking voluntary counselling and testing for HIV and sexually transmitted infections in Taiwan. *J Int AIDS Soc* 2014; **17**: 19640–19640.

50 Ho S-Y, Su L-H, Sun H-Y, *et al.* Trends of recent hepatitis C virus infection among HIV-positive men who have sex with men in Taiwan, 2011–2018. *eClinicalMedicine* 2020; **24**. DOI:10.1016/j.eclinm.2020.100441.

51 Yu M-L, Dai C-Y, Huang J-F, *et al.* Rapid virological response and treatment duration for chronic hepatitis C genotype 1 patients: a randomized trial. *Hepatology* 2008; **47**: 1884–93.

52 Huang C-F, Yeh M-L, Huang J-F, *et al.* Host interleukin-28B genetic variants versus viral kinetics in determining responses to standard-of-care for Asians with hepatitis C genotype 1. *Antiviral research* 2012; **93**: 239–44.

53 Yu M-L, Liu C-H, Huang C-F, *et al.* Revisiting the stopping rule for hepatitis C genotype 1 patients treated with peginterferon plus ribavirin. *PLoS one* 2012; **7**: e52048.
